# FXYD5/Dysadherin, a Biomarker of Endometrial Cancer Myometrial Invasion and Aggressiveness: Its Relationship With TGF-β1 and NF-κB Pathways

**DOI:** 10.3389/fonc.2019.01306

**Published:** 2019-12-06

**Authors:** María José Besso, Marina Rosso, Lara Lapyckyj, Cristian Pablo Moiola, María Laura Matos, María Florencia Mercogliano, Roxana Schillaci, Jaume Reventos, Eva Colas, Antonio Gil-Moreno, Alejandra Wernicke, Roberto Orti, Mónica Hebe Vazquez-Levin

**Affiliations:** ^1^Laboratorio de Estudios de la Interacción Celular en Reproducción y Cáncer, Instituto de Biología y Medicina Experimental (IBYME; CONICET-FIBYME), Buenos Aires, Argentina; ^2^Biomedical Research Group in Gynecology, Vall d'Hebron Research Institute (VHIR), Universitat Autónoma de Barcelona, CIBERONC, Barcelona, Spain; ^3^Laboratorio de Mecanismos Moleculares de Carcinogénesis, Instituto de Biología y Medicina Experimental (IBYME; CONICET-FIBYME), Buenos Aires, Argentina; ^4^Gynecological Oncology Department, Vall Hebron University Hospital, CIBERONC, Barcelona, Spain; ^5^Hospital Italiano de Buenos Aires, Buenos Aires, Argentina

**Keywords:** endometrial cancer, E-cadherin, FXYD5, dysadherin, TGF-β1, NF-κB, CCL-2

## Abstract

**Objective:** Endometrial cancer (EC) is the second most common gynecological cancer worldwide. Myometrial invasion (MI) is a key event in EC dissemination. This study aimed to evaluate FXYD5/dysadherin (FXYD5/Dys) expression in EC tissue and uterine aspirate (UA) biopsies and to assess molecular/functional changes associated with its expression in cellular models.

**Methods:** FXYD5/Dys messenger RNA (mRNA) levels were determined in EC tissue and UA biopsies. FXYD5/Dys expression was evaluated in EC RNAseq data from The Cancer Genome Atlas (TCGA) and GENEVESTIGATOR tools. FXYD5/Dys impact on E-cadherin expression and cell behavior was assessed in EC Hec1a cells treated with transforming growth factor (TGF)-β1, stably transfected with ETV5, and transiently transfected with FXYD5/Dys small interfering RNA (siRNA) or pcDNA3-FXYD5/Dys plasmid.

**Results:** FXYD5/Dys was associated with EC aggressiveness, finding high mRNA levels in tumors depicting MI > 50%, Grade 3, and intermediate/high risk of recurrence. FXYD5/Dys was highly expressed at the tumor invasive front compared to the superficial area. Most results were recapitulated in UA biopsies. FXYD5/Dys modulation in Hec1a cells altered cell migration/adhesion and E-cadherin expression. TGF-β1 treatment of Hec1a cells induced FXYD5/Dys expression. TCGA-UCEC RNAseq analysis revealed a positive correlation between FXYD5/Dys, TGF-β1, and plasminogen activator inhibitor (PAI)-1 mRNA levels. FXYD5/Dys induced nuclear factor (NF)-κB pathway activation in Hec1a cells. FXYD5/Dys mRNA levels positively correlated with transcriptional activation of NF-κB p65-regulated genes. Survival analysis revealed patient segregation into low- and high-risk groups, the latter depicting the highest FXYD5/Dys, PAI-1, tumor necrosis factor (TNF)-α, and TGF-β1 mRNA levels and shorter survival rates.

**Conclusion:** FXYD5/Dys is a novel biomarker of EC progression related to TGF-β1 and NF-κB pathways that collectively promote tumor dissemination and result in poor patient prognosis.

## Introduction

Endometrial cancer (EC) is the second most common gynecological neoplasm and the fourth most frequent women cancer worldwide. Projections for the coming years show a trend toward an increased number of EC cases (1). Histopathologically, EC is classified into two subtypes: Type I or endometrioid carcinomas (EEC) and Type II or non-EEC (NEEC) (2), with EEC being about 80% of EC cases (3). Myometrial invasion (MI) is a key event in EC dissemination and a critical factor to define the risk of recurrence. While Stage IA tumors (International Federation of Gynecology and Obstetrics, FIGO, 2009 classification) display <50% MI, Stages IB–IV display >50% MI (deep MI), the latter associated with poor prognosis (5-years survival rate: Stage IA, 90%, Stages IB–IV, 78–21%) (4). Routine EC diagnosis involves preoperative anatomopathological endometrial biopsy evaluation coupled to imaging techniques, adjusted at evaluation of the surgical piece. Among 75% tumors preoperatively classified as early stage EC (FIGO Stage I), ~20% are surgically reclassified as advanced-stage EC (FIGO Stages II–IV). Moreover, 30% of EC cases are diagnosed when the tumor has invaded >50% of the myometrial wall (5). This classification is critical for therapeutic management and greatly impacts post-surgery patient morbidity. Currently, there are no established molecular biomarkers to determine deep MI and/or to assist in risk stratification in a sensitive, objective, and reproducible fashion.

Alterations in cell–cell adhesion and decreased E-cadherin expression have been reported in EC progression (6–8). The expression of E-cadherin and related molecules was previously characterized in an EC cellular model of MI, generated by overexpression of ETV5 transcription factor in Hec1a cells (HGE cells) (9). HGE cells showed lower E-cadherin levels and epithelial-to-mesenchymal transition (EMT)-related molecular changes, as well as higher migratory/invasive properties than control cells (10). Moreover, HGE cells depicted higher expression [messenger RNA (mRNA)/protein] of FXYD5/dysadherin (FXYD5/Dys) (10). FXYD5/Dys is a Type I transmembrane glycoprotein, a member of the FXYD protein family (11). High FXYD5/Dys protein expression has been reported in several solid tumors in association to decreased cell–cell adhesion (12) and low E-cadherin protein expression (13–16). In addition, other E-cadherin-independent mechanisms have been proposed; in particular, an association has been found between FXYD5/Dys expression, nuclear factor (NF)-κB pathway activation, and CCL-2 (NF-κB target gene) upregulation in breast and renal cancer cell lines (17, 18). Moreover, an autocrine loop involving FXYD5/Dys, NF-κB pathway activation, and tumor necrosis factor (TNF) receptor 1 (TNFR1) has been suggested (19). More recently, these evidences were confirmed in a lung cancer model, where FXYD5/Dys expression was associated with NF-κB pathway activation, CCL-2, interleukin (IL)-6, and TNF-α upregulation and consequent monocyte recruitment (20). Finally, FXYD5/Dys expression has been related to distant metastasis and poor prognosis in various tumors (12).

The present study aimed to characterize FXYD5/Dys mRNA levels in EC samples and to evaluate the impact of modulating its expression upon cell behavior in established EC cell models. We proposed a relationship between FXYD5/Dys expression and EC tumor progression/aggressiveness.

## Materials and Methods

### Chemicals

Chemicals were of analytical or tissue culture grade and purchased from Sigma-Aldrich (St. Louis, MO, USA). Molecular biology and electrophoresis reagents were from Thermo-Fisher Scientific (Carlsbad, CA, USA) or BioRad (Hercules, CA, USA). The following antibodies were used: anti FXYD5/Dys: a) D-2 (mouse monoclonal) and b) FL-178 (rabbit polyclonal) from Santa Cruz Biotechnology (SCBT; Santa Cruz, CA, USA); anti E-cadherin 610181 (mouse monoclonal; Becton Dickinson Biosciences, BD; San Diego, CA, USA); anti NF-κB p65 (rabbit monoclonal; C22B4, Cell Signaling, Danvers, MA, USA); anti phospho-NF-κB p65 (Ser536) (rabbit monoclonal; 93H1, Cell Signaling); anti IκB-α (rabbit polyclonal; #9242, Cell Signaling); anti β-Tubulin (mouse monoclonal; clone D66, Sigma-Aldrich); anti GAPDH (rabbit monoclonal; clone 14C10, Cell Signaling). Cy3-labelled anti-mouse or anti-rabbit secondary antibodies (Sigma-Aldrich) were used for immunocytochemistry. Horseradish peroxidase-conjugated antimouse (Vector Laboratories Inc., Burlingame, CA, USA) or antirabbit (Sigma-Aldrich) IgG were used as secondary antibodies in Western immunoblotting assays.

### Plasmids

The coding sequence of human FXYD5/Dys was cloned in the pcDNA3 commercial plasmid (Thermo-Fisher Scientific) to generate the pcDNA3-FXYD5/Dys plasmid; the whole sequence was obtained by PCR amplification from a complementary DNA generated by retrotranscription of total RNA from MDA-MB-231 human breast cancer cells, followed by digestion with *Eco*RI and *Hin*dIII restriction enzymes, insert purification, and plasmid ligation. Nucleotide sequence analysis was done to confirm correct insertion and insert coding sequence.

### Patient Samples

Endometrial tissue samples were obtained from EC patients who underwent surgery before receiving hormonal and/or chemotherapy treatment at Vall d'Hebron Hospital (Barcelona, Spain) and at Hospital Italiano from Buenos Aires (Buenos Aires, Argentina). In addition, uterine aspirate (UA) samples from EC patients were collected (Vall d'Hebron Hospital) (21). Both institutional review boards approved the protocol, and a written informed consent was signed by all patients. Samples were classified based on the 2009 FIGO staging system. Sample collection and handling were done as previously described (21). Supplementary Tables 1–3 show patient sample clinical information.

### Cell Culture

Hec1a and HGE cells were cultured in McCoy's 5A medium (Thermo-Fisher Scientific), supplemented with 10% fetal bovine serum (FBS), as previously described (10).

### EC Cell Line Treatment With Recombinant Human TGF-β1

Hec1a cells (3 × 10^5^ cells per well of six-well plates) were cultured overnight, followed by treatment with 10 ng/ml of TGF-β1 (Tonbo Biosciences, San Diego, CA, USA) in a culture medium without FBS or vehicle (phosphate buffer saline, PBS). After 24 h, cells were processed for total RNA/protein extraction.

### EC Cell Line Transfection Studies

Downregulation of FXYD5/Dys expression in HGE was achieved by transient transfection of a FXYD5/Dys small interfering RNA (siRNA) (#45745, three target-specific 19–25-nt siRNAs designed to knock down gene expression; SCBT) with Lipofectamine® 2000 (Thermo-Fisher Scientific) following the protocol suggested by the manufacturer. Briefly, 5 × 10^5^ cells were seeded in each well of a sterile six-well plate and incubated overnight. Then, cells were transfected with 100 pmol/ml of FXYD5/Dys or control scramble siRNA (#37007, SCBT). In addition, Hec1a cells were transfected with pcDNA3-FXYD5/Dys or pcDNA3 (control) plasmids. Cells numbering 5 × 10^5^ were transfected with 1 μg of plasmid DNA with Lipofectamine® 2000. After a 5-h incubation period, the transfection mixture was replaced by a complete culture medium. Cells were subjected to transcript and protein expression analysis as well as to functional tests 24–72 h post-transfection, as specifically indicated.

### RNA Extraction, cDNA Synthesis, and Quantitative Real-Time PCR

Procedures were done as previously reported (22), with some modifications. Total RNA was extracted from tissues and UA biopsies and cell lines using standard protocols with TRIzol®(Thermo-Fisher Scientific). Synthesis of complementary DNA (cDNA) was done with 1–2 μg of total RNA using the SuperScript™ III reverse transcriptase enzyme (Thermo-Fisher Scientific). Quantitative evaluation of mRNA levels was performed by real-time PCR (RT-qPCR) using SYBR Green® PCR Master Mix (Thermo-Fisher Scientific) with the CFX96 Touch™ unit (Bio-Rad). Transcript expression levels were determined as follows: 2^−ΔCt^, where ΔCt = Ct gene under study – Ct housekeeping gene. GAPDH (cell lines and EC tissues) or POLR2A (UA samples) was used as housekeeping genes. When indicated, transcript relative expression was calculated using an appropriate reference sample, according to the 2^−ΔΔCt^ method (ΔΔCt = ΔCt sample - ΔCt reference). Supplementary Table 4 lists the primers used in this study.

### Fluorescence Immunocytochemistry

Cell monolayers were fixed with 4% paraformaldehyde, treated with 0.1% Triton X-100, blocked with 4% bovine serum albumin (BSA) in PBS, and placed for 1 h with specific primary antibodies, followed by a 1-h incubation with secondary antibodies. Nuclear cell staining was done with Hoechst 33342 (Sigma-Aldrich). Cell preparations were analyzed in a Nikon C1 confocal laser microscope (Tokyo, Japan). Images were evaluated using the ImageJ software (Wright Cell Imaging Facility, Toronto, ON, Canada) (22).

### Sample Preparation, SDS-PAGE, and Western Immunoblotting

Cell lysates were prepared in a RIPA (radio-immunoprecipitation assay) buffer with protease inhibitors. Protein extracts (20 μg) were electrophoresed in 10 and 12% SDS-polyacrylamide gels and electrotransferred to nitrocellulose membranes (Amersham Hybond ECL, GE Healthcare, Buckinghamshire, UK). Membranes were blocked in PBS containing 5% non-fat milk for 1 h, incubated overnight at 4°C with specific primary antibodies diluted in blocking solution, and for 1 h with secondary antibodies (0.4 μg/ml in a blocking solution). Assays were developed with an ECL Western Blotting Detection Kit (ECL, GE Healthcare) chemiluminescence system. Densitometric analysis was done using the ImageJ software (22).

### Hanging Drop Assay

EC cells were forced to grow under independent anchorage conditions to promote the formation of cellular aggregates, as previously reported (22). EC cells were harvested, and 20-μl drops of 2,000 cells were plated in the lid of a p100 dish and cultured for up to 72 h. Images were taken at 24–72 h to evaluate cell morphology and then analyzed using the ImageJ software.

### Wound Healing Assay

EC cells were subjected to the wound healing assay (22). Briefly, 2 × 10^5^ cells per well were cultured overnight to reach 90% confluence. Cell monolayers were scratched with a pipette tip, washed with PBS to remove non-adhered cells, and incubated for 48 h in a culture medium without FBS. Images were taken at 0–48 h and analyzed using the ImageJ software to determine the percentage of wound healing closure: % wound healing closure = (Area t_0_ – Area t_f_)/At_0_) × 100, where Area t_0_ is the area at the initial time and Area t_f_ is the area at the final time.

### GENEVESTIGATOR® and Co-expression Analysis

GENEVESTIGATOR™ (https://genevestigator.com/gv/index.jsp) integrates manually curated gene expression data from public repositories to investigate gene regulation at the transcriptional level using a wide variety of experimental conditions (23). In this study, the GENEVESTIGATOR® co-expression tool was used to find co-regulated genes with FXYD5/Dys in three datasets: “samples” (61,060 samples), “cancer” (634 categories), and “perturbations” (4,760 perturbations), analyzed using the HS_AFFY_U133PLUS_2-0 array. A co-expression value was calculated by Pearson's correlation coefficient.

### Gene Expression Analysis of EC Samples

To evaluate the relationship between FXYD5/Dys mRNA levels, E-cadherin (mRNA and protein), and NF-κB pathway activation, transcriptomic, proteomic, and clinical data from The Cancer Genome Atlas Uterine Corpus Endometrioid Cancer study (TCGA-UCEC) were retrieved from the UCSC Xena repository (https://xenabrowser.net/).

For transcriptomic analysis, RSEM-normalized RNAseq data from Illumina GA (*N* = 381) and Illumina HiSeq (*N* = 201) were downloaded. In addition, replicate-base normalization (RBN)-normalized proteomic data from E-cadherin immunodetection using reverse phase protein array (RPPA) were retrieved (*N* = 440 samples).

To analyze the regulatory transcription factor (TF) impact of NF-κB p65 on EC gene expression patterns, data from GA (*N* = 313), GAV2 (*N* = 349), and HiSeqV2 (*N* = 155) platforms were retrieved and compiled.

### Statistical Analysis

All experiments were performed at least in triplicate. Results were expressed as mean ± standard error (SEM). A *P* < 0.05 was considered statistically significant. Variable distribution analysis was done using Shapiro–Wilk's normality test. Student's *t*-test and Mann–Whitney test were used to compare two groups. Analysis of variance (ANOVA) and the Kruskal–Wallis test were used for comparisons involving more than two experimental groups. In some cases, the Wilcoxon signed-ranked test was applied. Paired human samples were analyzed with paired Student's *t*-test. For correlation analysis, the Spearman correlation coefficient, ρ (rho), was used. ROC (receiver operating characteristic) analysis was performed to determine the potential of FXYD5/Dys mRNA in preoperative UA as a marker of deep MI, high grade, and intermediate/high risk of recurrence. For survival analysis, Kaplan–Meier curves were constructed, and differences between them were analyzed by a log-rank test. GraphPad Prism software, version 5.01 (GraphPad Software, San Diego, CA, USA), was used to do the analyses.

## Results

### Expression of FXYD5/Dys in EC Samples and Association With Clinicopathological Parameters

**Figure 1:**
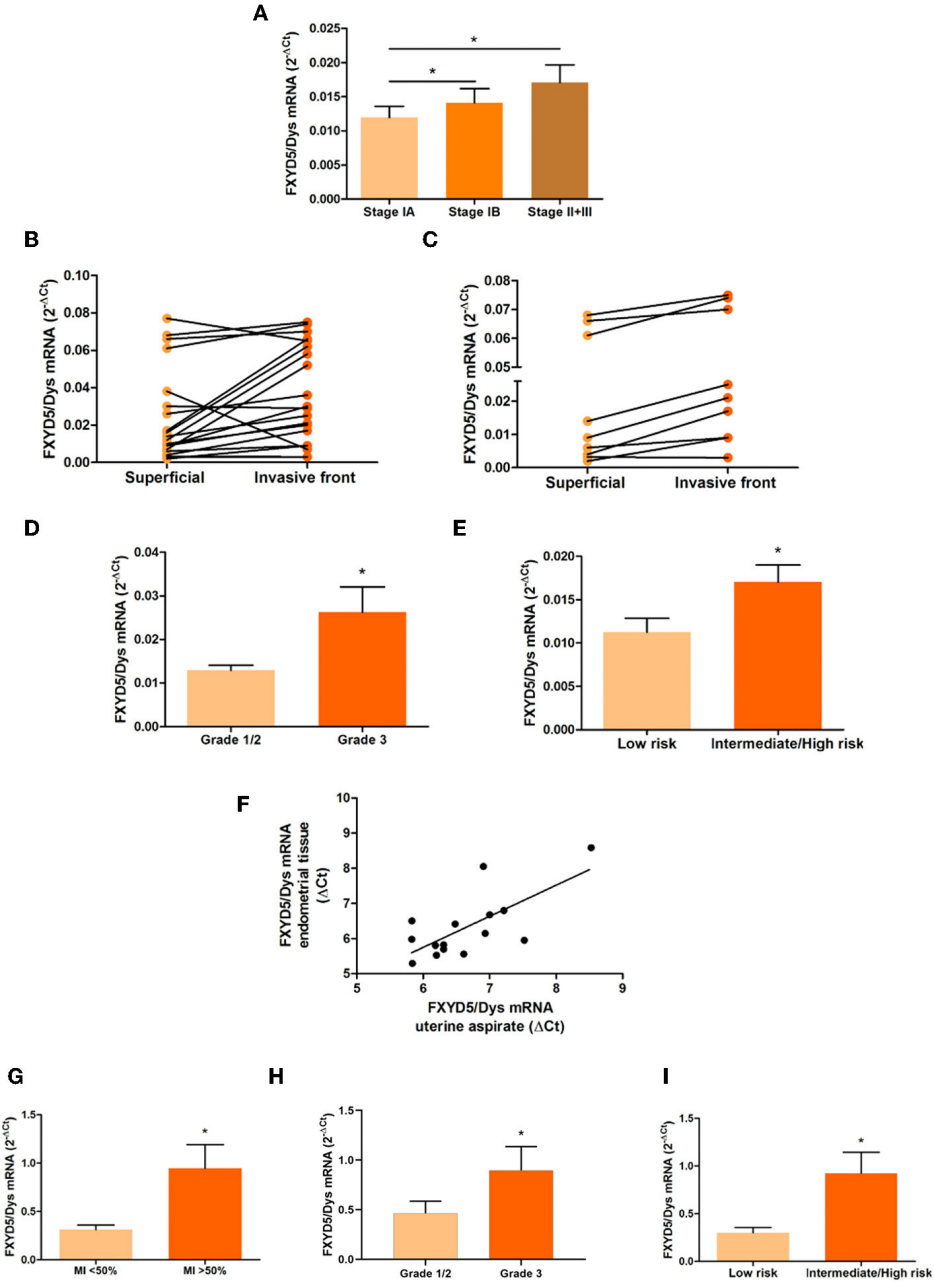
FXYD5/Dys mRNA expression and clinicopathological parameters in EC samples. **(A–C)** RT-qPCR analysis of FXYD5/Dys mRNA levels in **(A)** EEC samples grouped according to FIGO stage (Stage IA, *N* = 27; Stage IB, *N* = 15; and Stages II + III, *N* = 15) (differences observed between Stage IA and Stage IB tumors and between Stage IA and Stage II–III tumors; ^*^P < 0.05, ANOVA with Bonferroni post-test), **(B)** paired biopsies from superficial and invasive front of EEC samples (FIGO Stages I–III) (*N* = 20; *P* = 0.0123, paired *t*-test), and **(C)** paired biopsies from superficial and invasive front of Stage I EEC samples (*N* = 9; *P* = 0.0013, paired *t*-test). **(D)** FXYD5/Dys mRNA levels in Grade 1, 2, and 3 EEC samples (Stages I–III). EEC tumors classified as Grades 1 and 2 were included in the same group (Grade 1/2). Differences observed between Grade 1/2 tumors (*N* = 43) and Grade 3 tumors (*N* = 17) (*P* = 0.0381; unpaired *t*-test with Welch's correction). **(E)** RT-qPCR analysis of FXYD5/Dys expression in EC samples grouped according to risk of lymph node involvement and recurrence (low risk *N* = 19, intermediate/high risk *N* = 32; *P* = 0.0261, unpaired *t*-test with Welch's correction). **(F)** FXYD5/Dys mRNA levels assessed in UA biopsies. Correlation analysis between mRNA levels detected in uterine aspirate biopsies and paired EC tissue (*N* = 15; *r* = 0.5321, Spearman correlation, *P* = 0.0412). **(G–I)** RT-qPCR analysis of FXYD5/Dys expression in UA from EEC grouped according to **(G)** MI depth (MI < 50%, *N* = 11; MI > 50%, *N* = 10; *P* = 0.0315, unpaired *t*-test, with Welch's correction), **(H)** tumor grade (Grades 1 and 2, *N* = 13; Grade 3, *N* = 6; *P* = 0.0365, Mann–Whitney test), and **(I)** risk of lymph node involvement and recurrence (low risk, *N* = 10; intermediate/high risk, *N* = 11; *P* = 0.0190, unpaired *t*-test with Welch's correction).

FXYD5/Dys transcript levels were determined by RT-qPCR in EC tissue samples. As shown in Figure 1A, Stage IB as well as Stage II–Stage III tumors showed higher FXYD5/Dys mRNA levels than Stage IA tumors (*P* < 0.05). In line with these findings, in samples from the tumor invasive front, increased FXYD5/Dys mRNA levels were found compared with paired superficial samples (14/20, 70%; *P* = 0.0123) (Figure 1B). Among Stage I tumors, higher FXYD5/Dys mRNA levels were found at the invasive front compared to the superficial section of the tumor (*P* = 0.0013) (Figure 1C).

With regard to tumor grade, FXYD5/Dys mRNA expression levels were higher in Grade 3 than in Grade 1 and Grade 2 tumors (*P* = 0.0381) (Figure 1D). Based on these findings, the relationship between FXYD5/Dys transcript levels and the European Society for Medical Oncology (ESMO) risk stratification system (24) was assessed, finding higher FXYD5/Dys mRNA levels in intermediate/high-risk tumors than in low-risk ones (*P* = 0.0261) (Figure 1E).

A few years ago, UA biopsies became of interest in the evaluation of EC molecular biomarkers. Compared to conventional tissue biopsies, UA are a reliable source for EC biomarker assessment with high specificity and sensitivity, capable of capturing intra-tumor heterogeneity with a low-cost ambulatory sampling method (21, 25, 26). In order to evaluate FXYD5/Dys's potential as an MI preoperative biomarker, FXYD5/Dys expression levels were evaluated in preoperative UA biopsies from EC patients. First, a positive significant correlation (*r* = 0.5321; *P* = 0.0412) was found between FXYD5/Dys mRNA levels detected in UA and in tissue-paired biopsies (Figure 1F). Next, and in line with the results obtained from tissue biopsies, higher FXYD5/Dys mRNA levels were detected in UA biopsies from tumors depicting MI > 50% (*P* = 0.0315) (Figure 1G), Grade 3 (*P* = 0.0365) (Figure 1H), and intermediate/high risk of recurrence (*P* = 0.0190) (Figure 1I), compared to those with MI < 50%, Grades 1 and 2, or low risk of recurrence, respectively. ROC analysis revealed FXYD5/Dys as a significant predictor of MI [area under the curve (AUC) = 0.7545; *P* = 0.0486, sensitivity = 70%, specificity = 81.82%], grade (AUC = 0.8077; *P* = 0.0353, sensitivity = 83.33%, specificity = 69.23%), and risk of recurrence (AUC = 0.8091; *P* = 0.0167, sensitivity = 72.73%, specificity = 80%) in UA. The estimated cutoff value of FXYD5/Dys expression was 0.4178. Supplementary Figure 1 shows ROC curves for the three clinicopathological parameters.

### FXYD5/Dys Expression and EC Cell–Cell Adhesion and Migration

Taking into account results presented in the previous section and that modulation of FXYD5/Dys expression has been associated with an aggressive cellular behavior in breast and renal cancer cellular models (17, 18, 27), the impact of its knockdown upon cell behavior was evaluated. The EC cellular model of MI HGE was used to perform these studies. FXYD5/Dys downregulation in HGE cells was confirmed at mRNA and protein levels by RT-qPCR and Western immunoblotting/fluorescent immunocytochemistry, respectively (Supplementary Figure 2).

**Figure 2:**
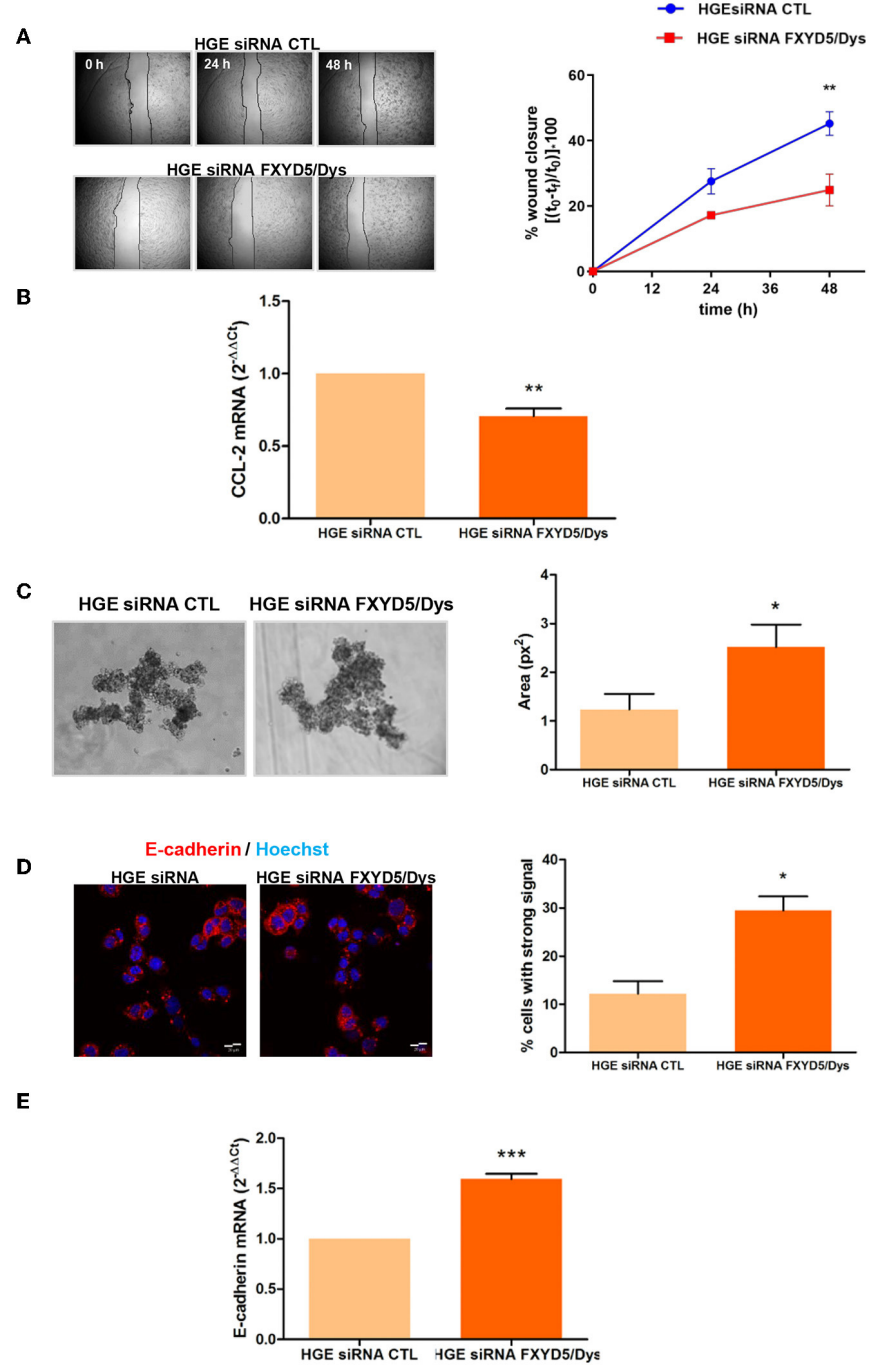
FXYD5/Dys regulation of cell–cell adhesion and cell migration in HGE cells. **(A)** Left panel: Wound healing assay of HGE siRNA FXYD5/Dys and HGE siRNA CTL cells. Representative images of cells at 0, 24, and 48 h are shown (magnification 40×). Right panel: Free-cell area quantified using ImageJ software. Wound healing closure percentage was plotted at every time point (**P < 0.01, two-way ANOVA, Bonferroni post-test). **(B)** RT-qPCR analysis of the pro-migratory chemokine CCL-2 expression in HGE siRNA CTL and HGE siRNA FXYD5/Dys cells (**P < 0.01, Wilcoxon signed rank test). **(C)** Left panel: Hanging drop assay of HGE siRNA FXYD5/Dys and HGE siRNA CTL cells. Representative images of cell aggregates at 72 h are shown (magnification 100×). Right panel: Cell aggregate area was quantified using ImageJ software, and mean cell aggregates areas (expressed as squared pixels) were plotted and compared (*P < 0.05, Mann–Whitney test). **(D)** Left panel: Fluorescent immunocytochemistry of HGE siRNA FXYD5/Dys and HGE siRNA CTR cell monolayers using anti-E-cadherin monoclonal antibody (610181; 2.5 μg/ml); cell nuclei are visualized using HOECHST 33342. Bar: 20 μm. Right panel: Percentage of cells depicting strong fluorescence intensity quantified using ImageJ software (*P < 0.05, Mann–Whitney test). **(E)** RT-qPCR analysis of E-cadherin mRNA in HGE siRNA FXYD5/Dys and HGE siRNA CTL cells (***P < 0.0001, Wilcoxon signed rank test).

Since FXYD5/Dys has been reported to promote cell motility *in vitro* in other tumor types (17, 18, 27), the impact of its knockdown in HGE cell migratory capacity was evaluated. HGE siRNA FXYD5/Dys cells depicted diminished migration compared to HGE siRNA CTL cells (*P* < 0.01) (Figure 2A). In line with these findings, HGE siRNA FXYD5/Dys cells showed a significant decrease in the promigratory chemokine CCL-2 mRNA levels when compared to HGE siRNA CTL cells (*P* < 0.01) (Figure 2B).

Studies done in breast cancer tissues showed a positive FXYD5/Dys immunostaining where cell–cell contacts had been lost (11). In the same study, FXYD5/Dys overexpression in liver cancer cell lines was found associated with decreased E-cadherin protein levels and decreased Ca^2+^-dependent cellular aggregation. Thus, the impact of FXYD5/Dys knockdown on HGE cell adhesiveness and E-cadherin expression was evaluated. Cellular aggregates formed by HGE siRNA FXYD5/Dys cells were larger than those in HGE siRNA CTL cells (*P* < 0.05) (Figure 2C). In line with this results, HGE siRNA FXYD5/Dys cells depicted higher E-cadherin expression than control cells, which was observed at protein (*P* < 0.05) (Figure 2D) and mRNA (*P* < 0.0001) levels (Figure 2E). Altogether, these results suggest a direct relationship between an HGE cell adhesive and migratory behavior and FXYD5/Dys expression.

**Figure 3:**
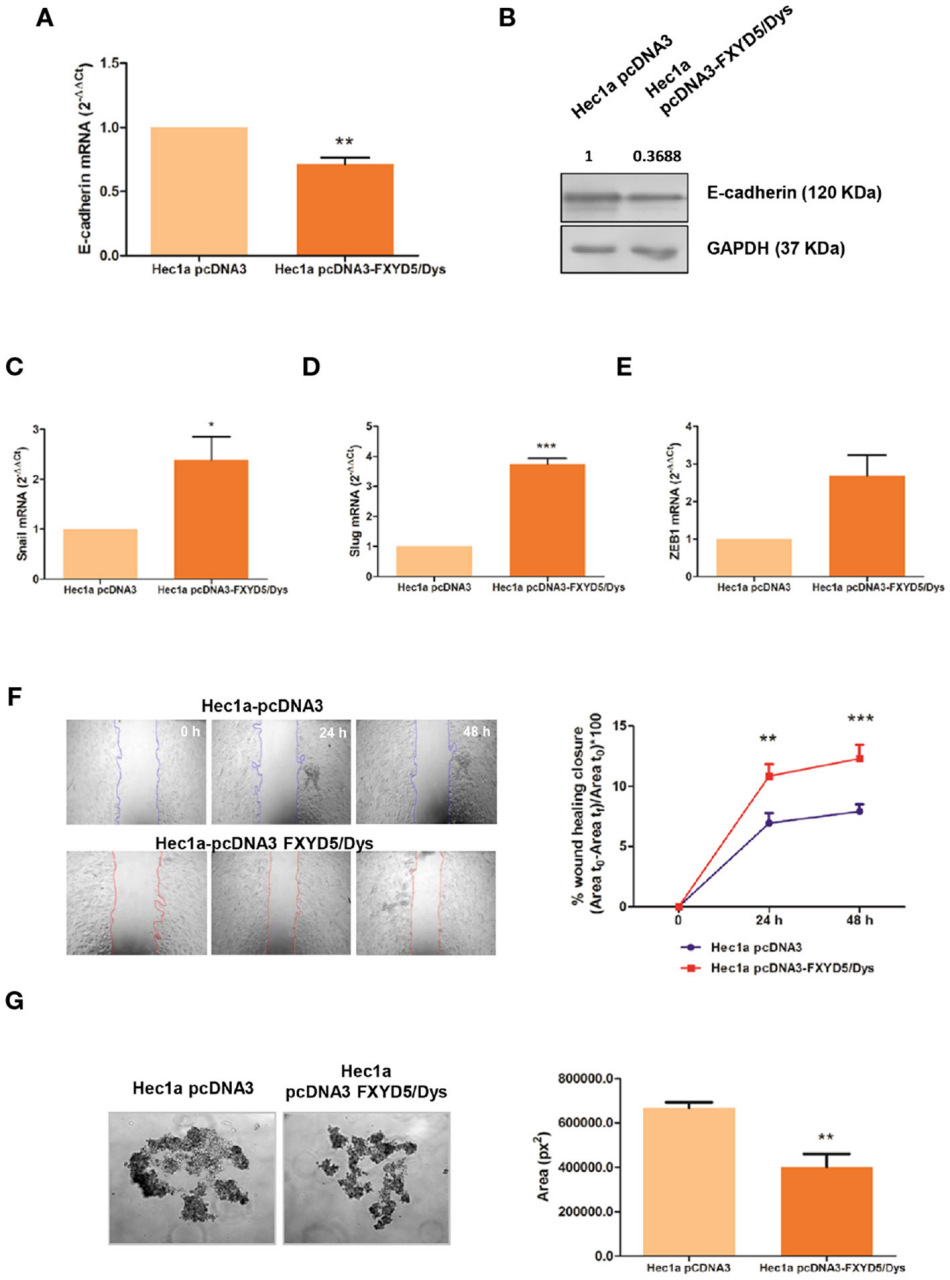
Modulation of FXYD5/Dys expression in Hec1a cells and changes in cell migration. **(A)** RT-qPCR analysis of E-cadherin mRNA expression in Hec1a cells transiently transfected with pcDNA3 empty plasmid (Hec1a pcDNA3 cells) or pcDNA3-FXYD5/Dys plasmid (Hec1a pcDNA3-FXYD5/Dys cells) (**P<0.01, Wilcoxon Signed Rank Test). **(B)** Immunodetection of E-cadherin by Western immunoblotting of Hec1a pcDNA3 and Hec1a pcDNA3-FXYD5/Dys cell protein extracts using anti E-cadherin monoclonal antibody (610181, BD; 0.125 μg/mL). GAPDH (anti GAPDH monoclonal antibody 14C10; 1:1000) was used as loading control. C-E. RT-qPCR analysis of E-cadherin transcriptional repressors Snail **(C)** (^*^P<0.05), Slug **(D)** (**P<0.01) and Zeb1 **(E)** (P=0.055), Wilcoxon Signed Rank Test). **(F)** Left panel: Wound healing assay of Hec1a pcDNA3 and Hec1a pcDNA3-FXYD5/Dys cells. Representative images of cells at 0, 24 and 48 h post transfection are shown (magnification 40X). Right panel: Free-cell area was quantified using ImageJ software and the wound healing closure percentage was plotted at every time point (**P<0.01, ***P<0.0001, Two-way ANOVA, Bonferroni post-test). **(G)** Left panel: Hanging drop assay of Hec1a pcDNA3 and Hec1a pcDNA3-FXYD5/Dys cells. Representative images of cell aggregates at 48h are shown (magnification 100X). Right panel: Cell aggregates area was quantified using ImageJ software and mean cell aggregates areas (expressed as squared pixels) were plotted and compared (**P<0.01, Mann Whitney test).

The effect of FXYD5/Dys expression upon cell behavior and E-cadherin expression was also evaluated in Hec1a cells that express high E-cadherin levels, by transient transfection with the pcDNA3-FXYD5/Dys plasmid (Hec1a pcDNA3-FXYD5/Dys). The transfection procedure effectiveness was verified by RT-qPCR and Western immunoblotting analysis (Supplementary Figure 3). FXYD5/Dys overexpression resulted in a decreased E-cadherin expression in Hec1a pcDNA3-FXYD5/Dys cells, evidenced by lower mRNA and protein levels (Figures 3A,B). In agreement with these results, an *in silico* analysis of the TCGA-UCEC RNAseq dataset (*N* = 397) showed a negative correlation between FXYD5/Dys mRNA and E-cadherin mRNA and protein levels (Supplementary Figure 4). These findings could be associated to E-cadherin transcriptional repression (28), as suggested by a higher expression of Snail (*P* < 0.05) and Slug (*P* < 0.01) and a trend for Zeb1 transcriptional repressors (Figures 3C–E) detected in Hec1a cells overexpressing FXYD5/Dys. With regard to cell behavior, FXYD5/Dys overexpression in Hec1a cells was associated with increased cell migration (*P* < 0.001) (Figure 3F) and decreased cell–cell adhesion, as evidenced by a decrease in Hec1a pcDNA3-FXYD5/Dys cell aggregate area (*P* < 0.001) (Figure 3G).

### Relationship Between FXYD5/Dys Expression and TGF-β1 in EC Cells

**Figure 4:**
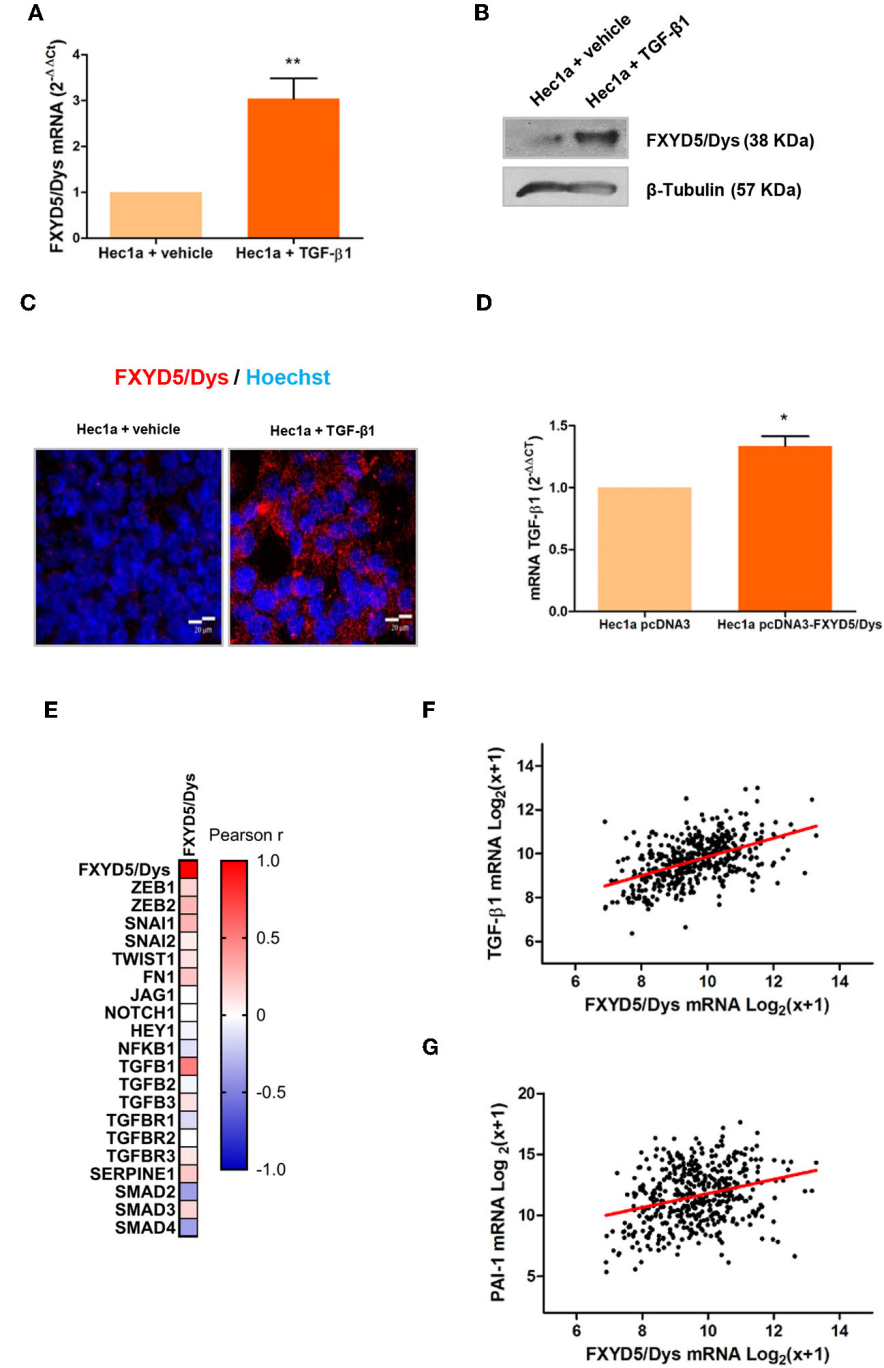
Association between TGF-β1 and FXYD5/Dys expression. **(A)** RT-qPCR analysis of FXYD5/Dys expression in Hec1a cells treated with 10 ng/ml TGF-β1 for 24 h. Controls were incubated with vehicle (**P < 0.01, Wilcoxon signed rank test). **(B)** Western immunoblotting of Hec1a protein extracts using FXYD5/Dys monoclonal antibody (sc-166782; 2 μg/ml). β-Tubulin was included as loading control (monoclonal antibody D66; 0.5 μg/ml). **(C)** Representative images of fluorescent immunocytochemistry analysis of FXYD5/Dys protein expression (anti-FXYD5/Dys polyclonal sc-98246; 2 μg/ml); cell nuclei were stained with HOECHST 33342, bar: 20 μm. **(D)** RT-qPCR analysis of TGF-β1 mRNA levels in Hec1a pcDNA3-FXYD5/Dys and Hec1a pcDNA3 cells (*P < 0.05, Wilcoxon signed rank test). **(E)** Correlation analysis between FXYD5/Dys and TGF-β pathway-related genes. A heatmap was built based on Pearson *r* correlation values for each gene. **(F)** Correlation analysis between TGF-β1 and FXYD5/Dys mRNA levels in EC samples from the TCGA-UCEC cohort (Pearson correlation, *r* = 0.4966, *N* = 444). mRNA levels are expressed as log_2_(*x* + 1) were *x* is the RSEM normalized expression value. **(G)** Correlation analysis between PAI-1 and FXYD5/Dys mRNA levels in EC samples from the TCGA-UCEC cohort (Pearson correlation, *r* = 0.2886, *N* = 444). mRNA levels are expressed as log_2_(*x* + 1) were *x* is the RSEM normalized expression value.

TGF-β-mediated signaling is activated in EC cells (29, 30) and plays a key role in the initial stages of invasion and metastasis (31). A co-expression analysis performed using the GENEVESTIGATOR database revealed that TGF-β1 was found between the transcripts most significantly co-expressed with FXYD5 in three independent-sample datasets (Supplementary Figures 5A–C, respectively). To determine the relationship between TGF-β1 and FXYD5/Dys expression, Hec1a cells were treated with human recombinant TGF-β1, and FXYD5/Dys expression was evaluated. As a result, FXYD5/Dys mRNA and protein levels increased in Hec1a upon TGF-β1 treatment (Figures 4A–C). On the other hand, Hec1a pcDNA3-FXYD5/Dys cells exhibited increased TGF-β1 mRNA levels compared with control cells (*P* < 0.05) (Figure 4D). Moreover, an RNAseq analysis from the TCGA-UCEC cohort revealed a positive correlation between FXYD5/Dys expression and several TGF-β signaling genes, including TGF-β1, ZEB1, ZEB2, SNAI1, FN1, SERPINE1 (PAI-1), and SMAD3 (Figure 4E). As an example, correlation analyses between FXYD5/Dys and TGF-β1 and between FXYD5/Dys and PAI-1 mRNA levels are shown (Figures 4F,G).

### FXYD5/Dys Expression and NF-κB Pathway Activation

FXYD5/Dys has been found to modulate NF-κB pathway activation (17–20). When it is taken into account that NF-κB has been suggested to play a key role during EC carcinogenesis (32, 33), a relationship between FXYD5/Dys and NF-κB activation in EC is found.

**Figure 5:**
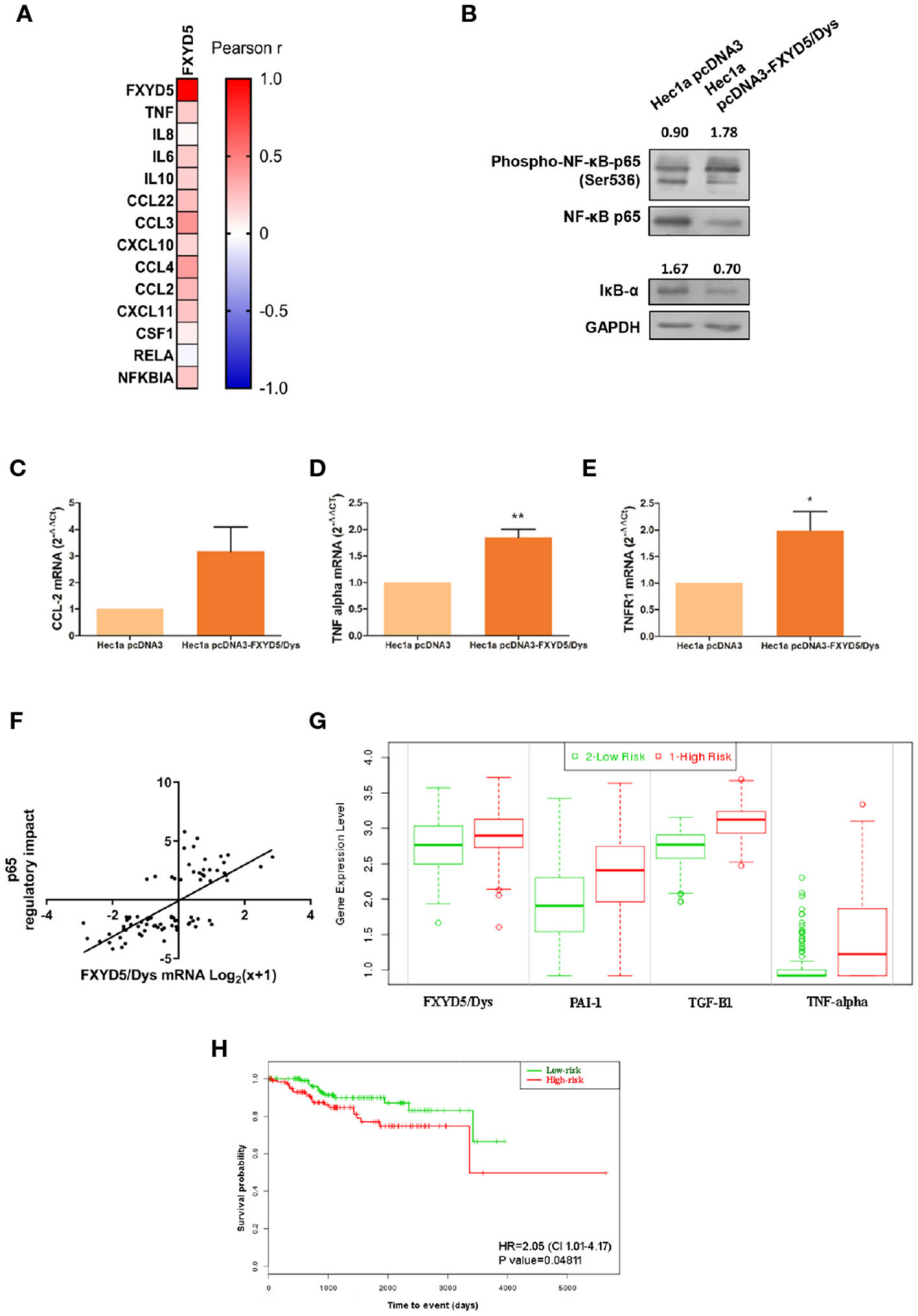
FXYD5/Dys expression and NF-κB pathway activation. **(A)** Correlation analysis between FXYD5/Dys and NF-κB pathway related genes. A heatmap was built based on Pearson r correlation values for each gene. **(B)** Evaluation of NF-κB pathway activation by Western immunoblotting. A representative image of p65 total and phosphorylated protein forms and IκB-α total protein form is shown. Antibodies: NF-κB p65 (monoclonal C22B4 # 4764, 1:1000 dilution), phospho-NF-κB p65 (Ser536) (monoclonal 93H1 # 3033, 1:1000 dilution) and IκB-α (polyclonal # 9242, 1:1000 dilution); GAPDH (anti GAPDH monoclonal antibody 14C10; 1:1000). C-E. RT-qPCR analysis of CCL-2 **(C)** (P=0.0654), TNF-α **(D)** (P<0.05) and TNFR1 **(E)** (P<0.01) (Wilcoxon signed-rank test) in Hec1a pcDNA3 and Hec1a pcDNA3-FXYD5/Dys cells. **(F)** Correlation analysis between FXYD5/Dys mRNA levels and transcriptional activation of genes regulated by p65. Regulatory impact of p65 transcription factor on tumor specific gene expression patterns from EC samples from the TCGA UCEC study. P65 regulatory impact to gene expression in each tumor is represented as a linear model tvalue, a positive t-value indicates p65 up-regulates its target genes and a negative t value indicates p65 down-regulates its target genes. FXYD5/Dys mRNA levels are expressed as Log_2_(x+1) were “x” is the RSEM normalized expression value. *** P<0.0001 (Spearman correlation, r=0.6927, N=81). G and **(H)** SurvExpress tool used to evaluate the impact of FXYD5/Dys, PAI-1, TGF-β1 and TNF alpha mRNA levels upon EC patients survival. This analysis was performed using gene expression and survival data of EC samples from the TCGA UCEC study (N=332). A Prognostic Index was estimated by beta coefficients multiplied by gene expression values of the four genes included in the analysis. Then, EC samples were divided in “Low Risk” and “High Risk” groups according to their Prognostic index values. **(G)** Box plots generated by SurvExpress showed the mRNA expression levels of FXYD5/Dys, PAI-1, TGF-β1 and TNF alpha. The P value from a *t* test of the difference between Low and High Risk groups was also calculated for each gene; FXYD5/Dys P= 3.80 e-03; SERPINE1 P= 2.05 e-08; TGF-β1 P=1.47 e-24; TNF P=3.79 e-10. Low-risk was in green and High-risk was in red, respectively. **(H)** Kaplan-Meier survival curves were constructed to evaluate the impact of FXYD5/Dys, PAI-1, TGF-β1, and TNF alpha mRNA levels (Low and High Risk groups) upon EC patient survival (^*^P<0.05, Log-Rank test).

First, RNAseq data analysis from the TCGA-UCEC cohort revealed a positive correlation between FXYD5/Dys and most of the NF-κB pathway genes evaluated (Figure 5A). Moreover, experiments performed in Hec1a cells revealed increased NF-κB (*P* < 0.001) and decreased total protein levels of the IκB-α inhibitor in Hec1a pcDNA3-FXYD5/Dys cells compared to Hec1a-pcDNA3 cells (Figure 5B). These changes were accompanied by a trend toward higher CCL-2 (*P* = 0.0654) expression and increased TNF-α (*P* = 0.0313) mRNA levels (Figures 5C,D), two target genes of the NF-κB pathway. Moreover, an increase in TNFR1 mRNA levels (*P* = 0.0362) was also observed (Figure 5E). In line with these results, a significant correlation was found between FXYD5/Dys mRNA levels and transcriptional activation of the NF-κB p65-regulated genes in EC samples from the TCGA-UCEC study (Figure 5F) and an increased expression of several NF-κB pathway target genes, among them CCL-2 and TNF-α (Supplementary Table 5).

Finally, to assess the prognostic significance of FXYD5/Dys expression and genes related to TGF-β and NF-κB pathways, a survival analysis was done in the TCGA-UCEC study using the SurvExpress tool. As a result of this analysis, two risk groups (low and high risk) were defined based on the mRNA expression levels of FXYD5/Dys, TGF-β1, PAI-1, and TNF-α (Figure 5G). Patients included in the high-risk group were characterized by an increased expression of the four genes and a significant lower (*P* < 0.05) survival rate (Figure 5H).

## Discussion

An increased expression of FXYD5/Dys has been related to metastasis and poor prognosis in several tumors. In some tumors and cell lines, FXYD5/Dys overexpression significantly correlated with a decreased E-cadherin expression, leading authors to propose that FXYD5/Dys would promote metastasis through a negative modulation of E-cadherin expression/functions (13–16). In addition, FXYD5/Dys expression has been shown to induce *in vitro* changes in cell morphology and to *in vivo* promote metastasis in cell lines and tumors that do not express E-cadherin (17). The present study aimed to characterize FXYD5/Dys expression in EC. RT-qPCR studies in EC tissue samples revealed an association between increased FXYD5/Dys mRNA levels and several EC clinicopathological parameters. Among them, tumors with deep MI showed higher FXYD5/Dys mRNA levels, even in Stage I tumors. Moreover, the tumor invasive front had higher FXYD5/Dys mRNA levels than the superficial tumor section, suggesting an involvement of FXYD5/Dys in tumor dissemination. In addition, FXYD5/Dys expression was higher in Grade 3 tumors than in Grade 1 and Grade 2 tumors, as observed in other cancers (34, 35). Consequently, intermediate/high-risk tumors showed higher FXYD5/Dys mRNA levels than low-risk tumors. In addition, FXYD5/Dys mRNA levels detected in preoperative UA reproduced tissue biopsy results and demonstrated the diagnostic potential of FXYD5/Dys transcript evaluation for MI, grade, and risk of recurrence.

Results on FXYD5/Dys expression in EC patient samples suggest its role in EC progression. To further address this hypothesis, modulation of FXYD5/Dys expression was done in EC cell models. Firstly, FXYD5/Dys downregulation in HGE cells resulted in lower cell migration and decreased CCL-2 mRNA levels, in agreement with previous findings (17, 18). Also, FXYD5/Dys knockdown was associated with an increase in HGE cell–cell adhesiveness, evidenced in the formation of larger cell aggregates and increased E-cadherin expression, suggesting an inverse relationship between both proteins. On the other hand, FXYD5/Dys overexpression in Hec1a cells resulted in increased cell migration and decreased cell adhesive capacity; at the molecular level, these changes were associated with a decreased E-cadherin expression, accompanied by increased mRNA levels of Snail, Slug, and ZEB1, in agreement with previous reports in tumor samples (6, 36). The results obtained after Hec1a EC cell modulation of FXYD5/Dys expression are in line with results obtained in EC patient samples showing higher FXYD5/Dys transcript levels in invasive EC (MI > 50%) and at the invasive tumor front, as well as in Grade 3 tumors. Altogether, these findings lead us to propose that an increased FXYD5/Dys expression could promote cell migration and dedifferentiation, in part through E-cadherin downregulation, in EC cells, thus facilitating MI and tumor dissemination.

A co-expression analysis using three global expression studies identified TGF-β1 as one of the molecules most significantly co-expressed with FXYD5/Dys. Moreover, TGF-β1 induced an increase in FXYD5/Dys expression in Hec1a cells, and FXYD5/Dys overexpression increased TGF-β1 expression. Furthermore, an association was found between elevated mRNA levels of FXYD5/Dys and several TGF-β genes in the TCGA-UCEC cohort. Altogether, these results lead us to propose an autocrine and/or paracrine regulation loop between FXYD5/Dys and the TGF-β1 pathway, in which TGF-β1 produced by the tumor microenvironment or by tumor cells could induce FXYD5/Dys expression in the tumor cell. FXYD5/Dys could then activate TGF-β1 expression, leading to sustained pathway activation.

Our results revealed that overexpression of FXYD5/Dys in Hec1a cells induced the NF-κB pathway, leading to activation of NF-κB target genes, as well as of TNFR1, in line with previous studies (19). In addition, TCGA RNAseq data analysis revealed a correlation between FXYD5/Dys mRNA levels with transcriptional activation of NF-κB p65-regulated genes in EC. FXYD5/Dys expression has been associated with NF-κB pathway activation in cancer models (17), and more recently, it has been shown to consequently induce macrophage/monocyte recruitment mediated by CCL-2/CCR2 (20). Elevated infiltration of macrophages (37, 38) and lymphocytes (39–42) has been found in EC tissues compared to normal tissues. Moreover, macrophage infiltration was associated with poor prognosis in EC (37, 38, 43). Also, low preoperative LMR (lymphocyte–monocyte ratio) values were associated with shorter survival rates (44). Moreover, high monocyte counts were associated with advanced stage, deep MI, lymph node metastases, and decreased survival at the time of recurrence (45). An RNAseq data analysis performed by us on the TCGA-UCEC cohort revealed a positive correlation between FXYD5/Dys and CD68 (monocyte/macrophage marker) (*P* < 0.0001) and CD163 (macrophage activation marker) (*P* < 0.0001) (data not shown). Altogether, these results lead us to propose FXYD5/Dys as a potential inflammation mediator in EC, facilitating monocyte recruitment to the tumor microenvironment and eventually promoting cancer progression, through NF-κB pathway activation.

Survival analysis revealed the potential use of the combined expression of FXYD5/Dys, PAI-1, TGF-β1, and TNF-α transcripts as a predictor of EC patient survival outcome. However, even when the results showed statistical significance, an independent survival analysis should be carried out in an independent cohort of patients in order to obtain more robust findings. One possible cause of these findings may relate to the disparity in the percentages of death and censored events in the cohort analyzed. The population included 247 EC patients, from which 33 (13%) experienced the event (death) and 214 (87%) were classified as censored events. Moreover, the clinicopathological characteristics of the cohort showed a predominance of tumors depicting favorable prognostic factors, evidenced by 80% of cases with endometrioid histology, 66% with FIGO Stage I tumors, 62% with tumors having MI < 50%, and 62% with histologic Grade 1 or 2. Since our findings demonstrate an association between higher FXYD5/Dys mRNA levels and advanced FIGO stage, deep MI, and high histological grade, the survival analysis results could be justified, at least in part, by the characteristics of the studied cohort. However, additional molecular mechanisms underlying the EC pathogenesis (FXYD5/Dys-related and non-related) should not be ruled out. Thus, FXYD5/Dys would exert its action through various molecular mechanisms highly associated with EC progression, which has a negative impact on patients' prognosis.

Altogether, results here presented lead us to propose FXYD5/Dys as a biomarker of EC progression and aggressiveness. These findings are the first evidence of FXYD5/Dys implication in EC progression. The evaluation of FXYD5/Dys expression may contribute to current tools for EC management, in addition to deepening the molecular basis of EC. Additional prospective studies will further help in confirming the diagnostic/prognostic value of FXYD5/Dys in EC.

## Data Availability Statement

The datasets generated for this study can be found in the manuscript.

## Ethics Statement

Both Institutional Ethical Review Boards approved the protocol (Vall d'Hebron Hospital Protocol SAF2008_3997; Hospital Italiano Protocol #1895) and a written informed consent signed by all patients; the IBYME institutional Ethical Committee reviewed and accepted both clinical protocols (CE021-2/2012).

## Author Contributions

MB and MV-L were involved in the study conception and design. MB, MR, LL, CM, MLM, MFM, RS, EC, AG-M, AW, RO, and MV-L participated in the data collection. MB, MR, and MV-L participated in the data analysis. JR and MV-L were responsible for the grants that supported this study. MB and MV-L drafted the manuscript. All authors read and accepted the final version. MV-L was responsible for coordinating and supervising the entire project.

### Conflict of Interest

The authors declare that the research was conducted in the absence of any commercial or financial relationships that could be construed as a potential conflict of interest.
