# Corrigendum: FXYD5/Dysadherin, a biomarker of endometrial cancer myometrial invasion and aggressiveness: its relationship with TGF-β1 and NF-κB pathways

**DOI:** 10.3389/fonc.2023.1322204

**Published:** 2024-01-23

**Authors:** María José Besso, Marina Rosso, Lara Lapyckyj, Cristian Pablo Moiola, María Laura Matos, María Florencia Mercogliano, Roxana Schillaci, Jaume Reventos, Eva Colas, Antonio Gil-Moreno, Alejandra Wernicke, Roberto Orti, Mónica Hebe Vazquez-Levin

**Affiliations:** ^1^ Laboratorio de Estudios de la Interacción Celular en Reproducción y Cáncer, Instituto de Biología y Medicina Experimental (IBYME; CONICET-FIBYME), Buenos Aires, Argentina; ^2^ Biomedical Research Group in Gynecology, Vall d’Hebron Research Institute (VHIR), Universitat Autónoma de Barcelona, CIBERONC, Barcelona, Spain; ^3^ Laboratorio de Mecanismos Moleculares de Carcinogénesis, Instituto de Biología y Medicina Experimental (IBYME; CONICET-FIBYME), Buenos Aires, Argentina; ^4^ Gynecological Oncology Department, Vall Hebron University Hospital, CIBERONC, Barcelona, Spain; ^5^ Hospital Italiano de Buenos Aires, Buenos Aires, Argentina

**Keywords:** endometrial cancer, E-cadherin, FXYD5, dysadherin, TGF-β1, NF-κB, CCL-2

In the published article, there was an omission in **Materials and Methods.** Anti GAPDH monoclonal antibody 14C10 was used as loading control in experiments presented in **Figures 3**, **5**. The correct materials and methods paragraph appears below.

**Figure 3:**
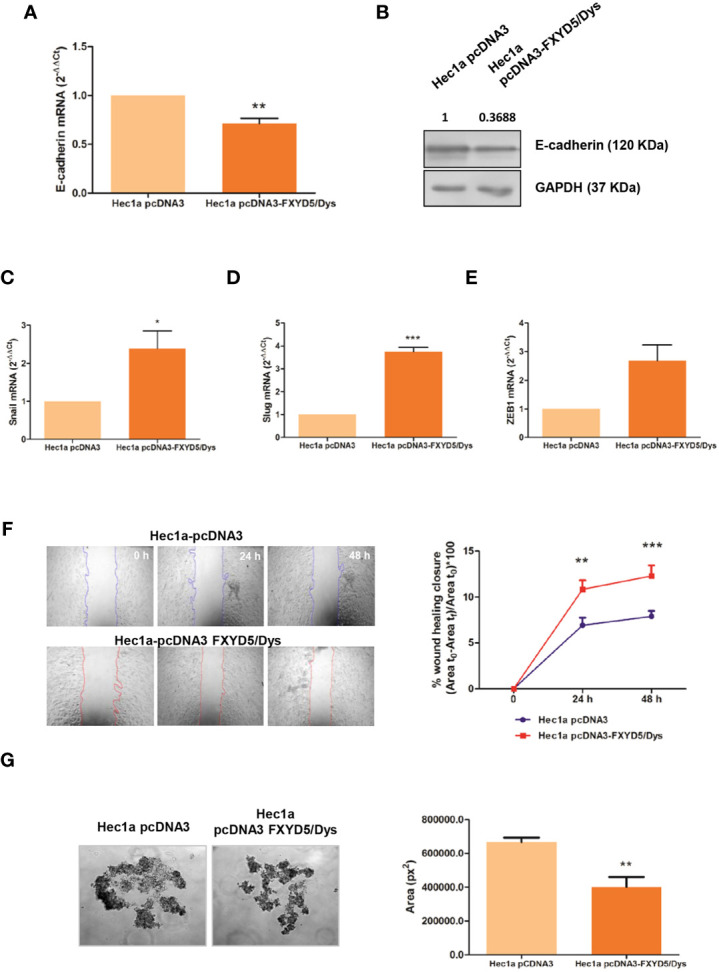
corrected. Modulation of FXYD5/Dys expression in Hec1a cells and changes in cell migration. **(A)** RT-qPCR analysis of E-cadherin mRNA expression in Hec1a cells transiently transfected with pcDNA3 empty plasmid (Hec1a pcDNA3 cells) or pcDNA3-FXYD5/Dys plasmid (Hec1a pcDNA3-FXYD5/Dys cells) (**P<0.01, Wilcoxon Signed Rank Test). **(B)** Immunodetection of E-cadherin by Western immunoblotting of Hec1a pcDNA3 and Hec1a pcDNA3-FXYD5/Dys cell protein extracts using anti E-cadherin monoclonal antibody (610181, BD; 0.125 µg/mL). GAPDH (anti GAPDH monoclonal antibody 14C10; 1:1000) was used as loading control. C-E. RT-qPCR analysis of E-cadherin transcriptional repressors Snail **(C)** (*P<0.05), Slug **(D)** (**P<0.01) and Zeb1 **(E)** (P=0.055), Wilcoxon Signed Rank Test). **(F)** Left panel: Wound healing assay of Hec1a pcDNA3 and Hec1a pcDNA3-FXYD5/Dys cells. Representative images of cells at 0, 24 and 48 h post transfection are shown (magnification 40X). Right panel: Free-cell area was quantified using ImageJ software and the wound healing closure percentage was plotted at every time point (**P<0.01, ***P<0.0001, Two-way ANOVA, Bonferroni post-test). **(G)** Left panel: Hanging drop assay of Hec1a pcDNA3 and Hec1a pcDNA3-FXYD5/Dys cells. Representative images of cell aggregates at 48h are shown (magnification 100X). Right panel: Cell aggregates area was quantified using ImageJ software and mean cell aggregates areas (expressed as squared pixels) were plotted and compared (**P<0.01, Mann Whitney test).

**Figure 5:**
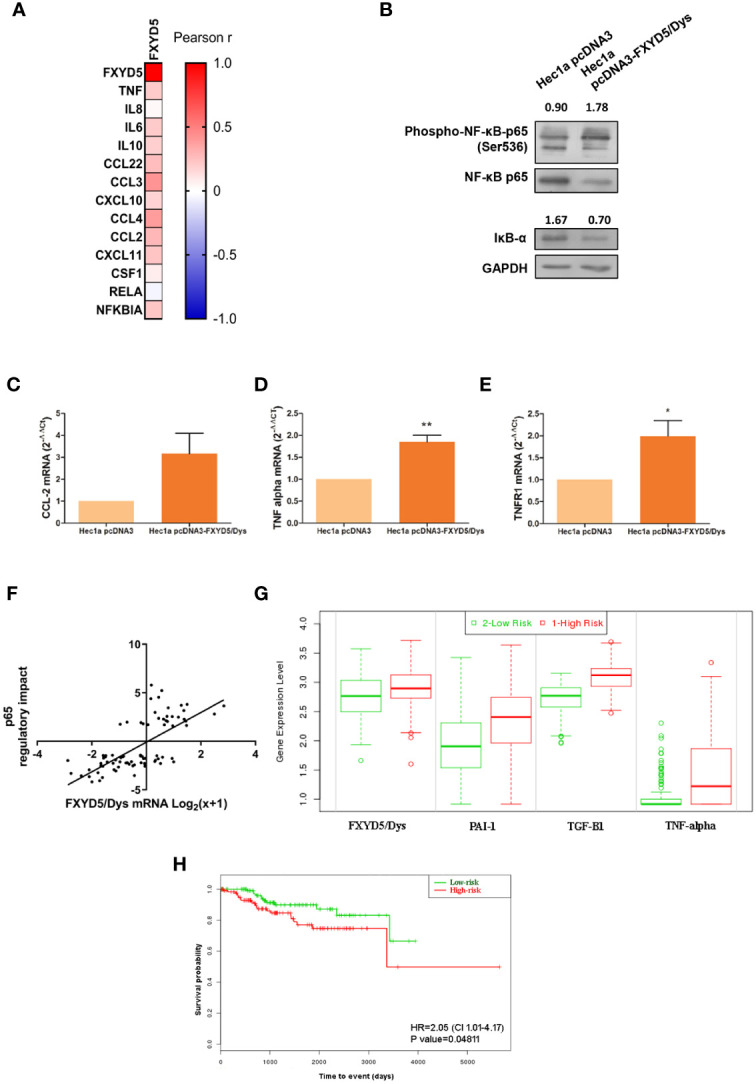
corrected. FXYD5/Dys expression and NF-κB pathway activation. **(A)** Correlation analysis between FXYD5/Dys and NF-κB pathway related genes. A heatmap was built based on Pearson r correlation values for each gene. **(B)** Evaluation of NF-κB pathway activation by Western immunoblotting. A representative image of p65 total and phosphorylated protein forms and IκB-α total protein form is shown. Antibodies: NF-κB p65 (monoclonal C22B4 # 4764, 1:1000 dilution), phospho-NF-κB p65 (Ser536) (monoclonal 93H1 # 3033, 1:1000 dilution) and IκB-α (polyclonal # 9242, 1:1000 dilution); GAPDH (anti GAPDH monoclonal antibody 14C10; 1:1000). C-E. RT-qPCR analysis of CCL-2 **(C)** (P=0.0654), TNF-α **(D)** (P<0.05) and TNFR1 **(E)** (P<0.01) (Wilcoxon signed-rank test) in Hec1a pcDNA3 and Hec1a pcDNA3-FXYD5/Dys cells. **(F)** Correlation analysis between FXYD5/Dys mRNA levels and transcriptional activation of genes regulated by p65. Regulatory impact of p65 transcription factor on tumor specific gene expression patterns from EC samples from the TCGA UCEC study. P65 regulatory impact to gene expression in each tumor is represented as a linear model t-value, a positive t-value indicates p65 up-regulates its target genes and a negative t value indicates p65 down-regulates its target genes. FXYD5/Dys mRNA levels are expressed as Log_2_(x+1) were “x” is the RSEM normalized expression value. *** P<0.0001 (Spearman correlation, r=0.6927, N=81). G and **(H)** SurvExpress tool used to evaluate the impact of FXYD5/Dys, PAI-1, TGF-β1 and TNF alpha mRNA levels upon EC patients survival. This analysis was performed using gene expression and survival data of EC samples from the TCGA UCEC study (N=332). A Prognostic Index was estimated by beta coefficients multiplied by gene expression values of the four genes included in the analysis. Then, EC samples were divided in “Low Risk” and “High Risk” groups according to their Prognostic index values. **(G)** Box plots generated by SurvExpress showed the mRNA expression levels of FXYD5/Dys, PAI-1, TGF-β1 and TNF alpha. The P value from a *t* test of the difference between Low and High Risk groups was also calculated for each gene; FXYD5/Dys P= 3.80 e-03; SERPINE1 P= 2.05 e-08; TGF-β1 P=1.47 e-24; TNF P=3.79 e-10. Low-risk was in green and High-risk was in red, respectively. **(H)** Kaplan-Meier survival curves were constructed to evaluate the impact of FXYD5/Dys, PAI-1,TGF-β1, and TNF alpha mRNA levels (Low and High Risk groups) upon EC patient survival (*P<0.05, Log-Rank test).

## 2 Materials and Methods

### 2.1 Chemicals

Chemicals were of analytical or tissue culture grade and purchased from Sigma-Aldrich (St. Louis, MO, USA). Molecular biology and electrophoresis reagents were from Thermo-Fisher Scientific (Carlsbad, CA, USA) or BioRad (Hercules, CA, USA). The following antibodies were used: anti FXYD5/Dys: a) D-2 (mouse monoclonal) and b) FL-178 (rabbit polyclonal) from Santa Cruz Biotechnology (SCBT; Santa Cruz, CA, USA); anti E-cadherin 610181 (mouse monoclonal; Becton Dickinson Biosciences, BD; San Diego, CA, USA); anti NF-κB p65 (rabbit monoclonal; C22B4, Cell Signaling, Danvers, MA, USA); anti phospho-NF-κB p65 (Ser536) (rabbit monoclonal; 93H1, Cell Signaling); anti IκB-α (rabbit polyclonal; #9242, Cell Signaling); anti β-Tubulin (mouse monoclonal; clone D66, Sigma-Aldrich); anti GAPDH (rabbit monoclonal; clone 14C10, Cell Signaling). Cy3-labelled anti-mouse or anti-rabbit secondary antibodies (Sigma-Aldrich) were used for immunocytochemistry. Horseradish peroxidase-conjugated anti-mouse (Vector Laboratories Inc., Burlingame, CA, USA) or anti-rabbit (Sigma-Aldrich) IgG were used as secondary antibodies in Western immunoblotting assays.

The authors apologize for this error and state that this does not change the scientific conclusions of the article in any way. The original article has been updated.

In the published article, there was an error in **Figure 3** **. Modulation of FXYD5/Dys expression in Hec1a cells and changes in cell migration,** and in **Figure 5**. **FXYD5/Dys expression and NF-κB pathway activation.** In both cases, it was indicated that “β-Tubulin (anti β-Tubulin monoclonal antibody D66; 0.5 µg/mL) was used as loading control”. This is a mistake, since in both cases anti GAPDH monoclonal antibody 14C10 was used as loading control.

The corrected Figure 3 and Figure 5 and their legends appear below.

The authors apologize for this error and state that this does not change the scientific conclusions of the article in any way. The original article has been updated.

