# Supplementary figures and images for: FXYD5/Dysadherin, a Biomarker of Endometrial Cancer Myometrial Invasion and Aggressiveness: Its Relationship With TGF-β1 and NF-κB Pathways
Source: Front Oncol. 2019 Dec 6;9:1306. doi: 10.3389/fonc.2019.01306 (PMC6908519; doi:10.3389/fonc.2019.01306)

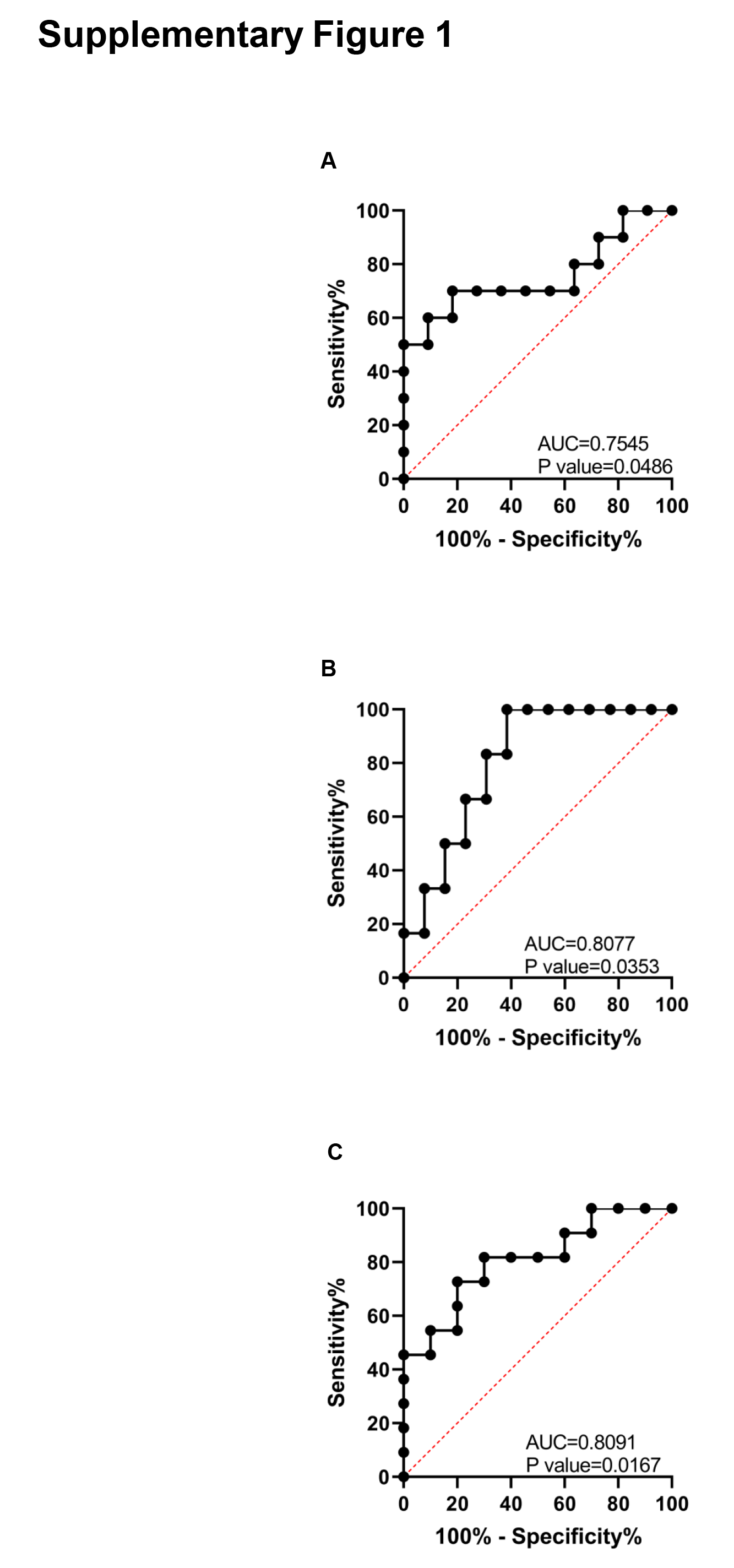

Supplement: Supplementary file 1 [file Data_Sheet_1.zip › SUPPL_FIGURE_1_BESSO ET AL.tif]

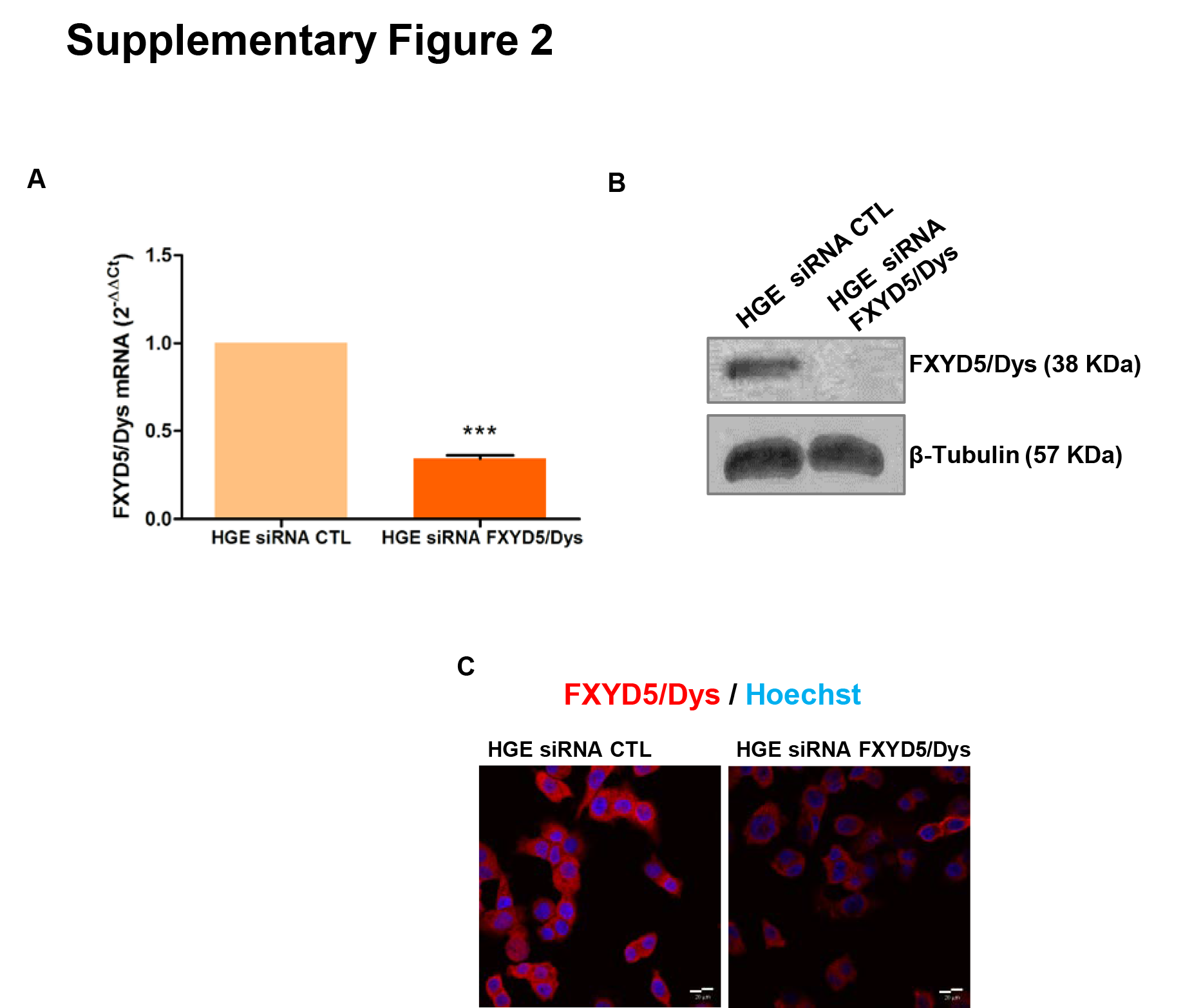

Supplement: Supplementary file 1 [file Data_Sheet_1.zip › SUPPL_FIGURE_2_BESSO ET AL.tif]

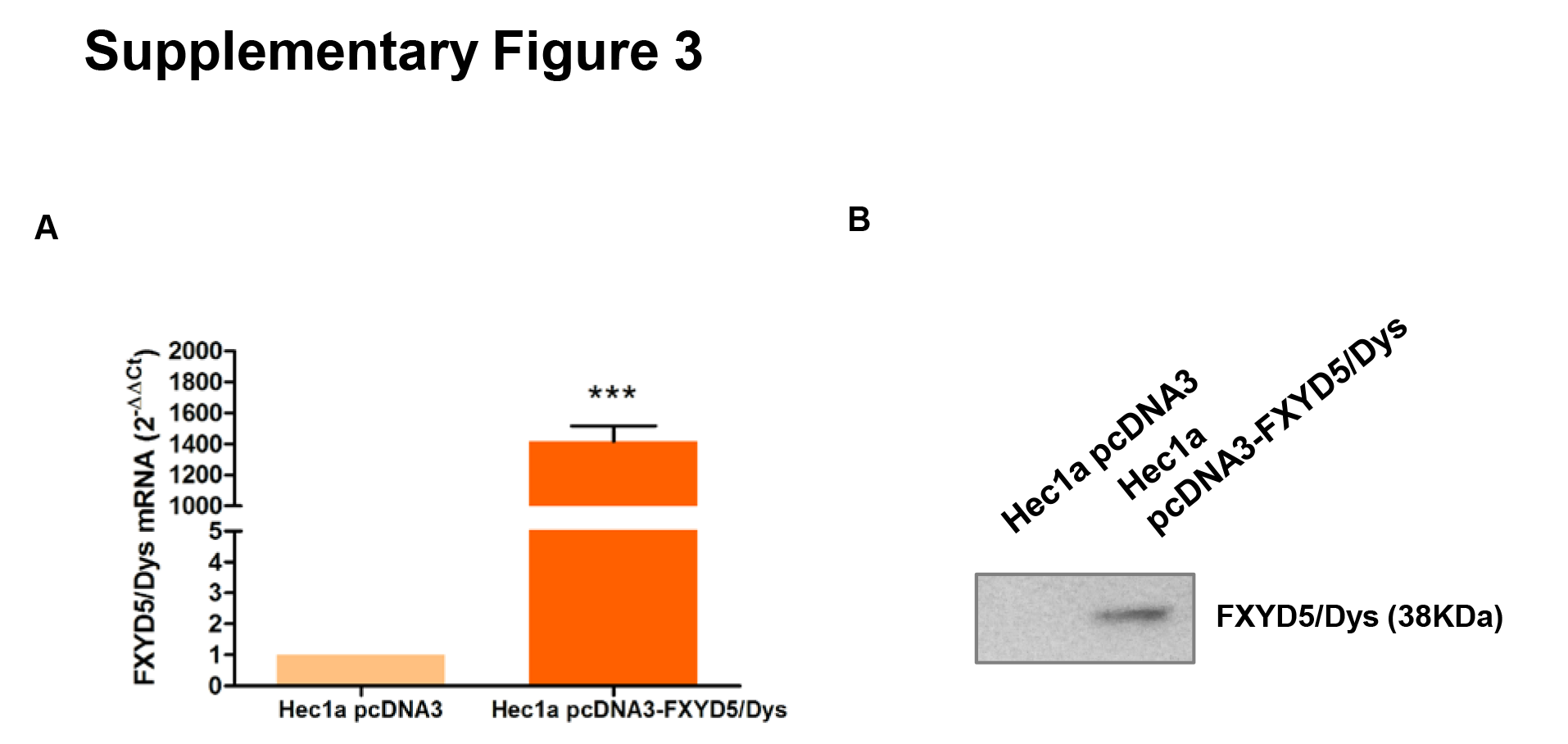

Supplement: Supplementary file 1 [file Data_Sheet_1.zip › SUPPL_FIGURE_3_BESSO ET AL.tif]

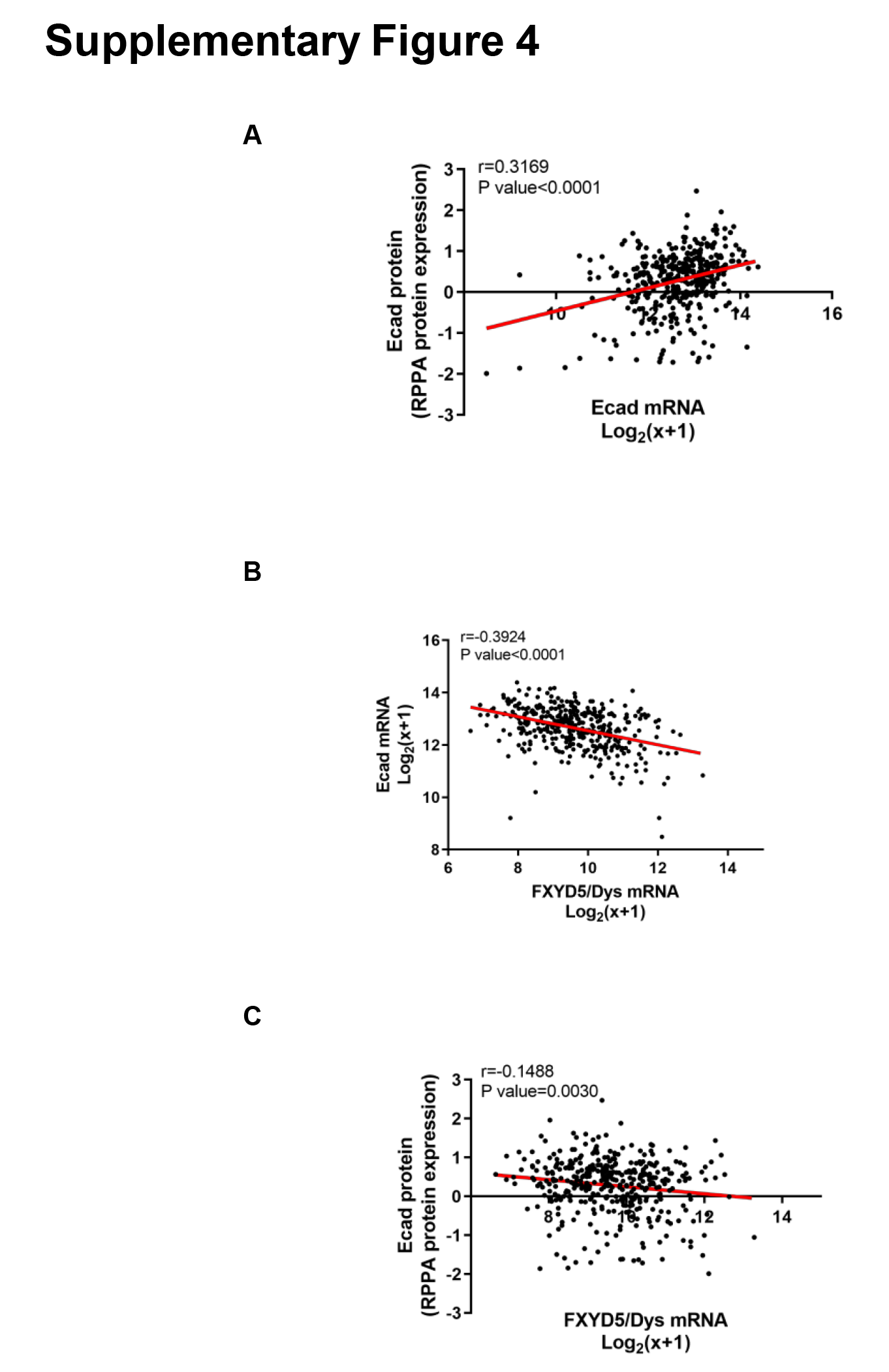

Supplement: Supplementary file 1 [file Data_Sheet_1.zip › SUPPL_FIGURE_4_BESSO ET AL.tif]

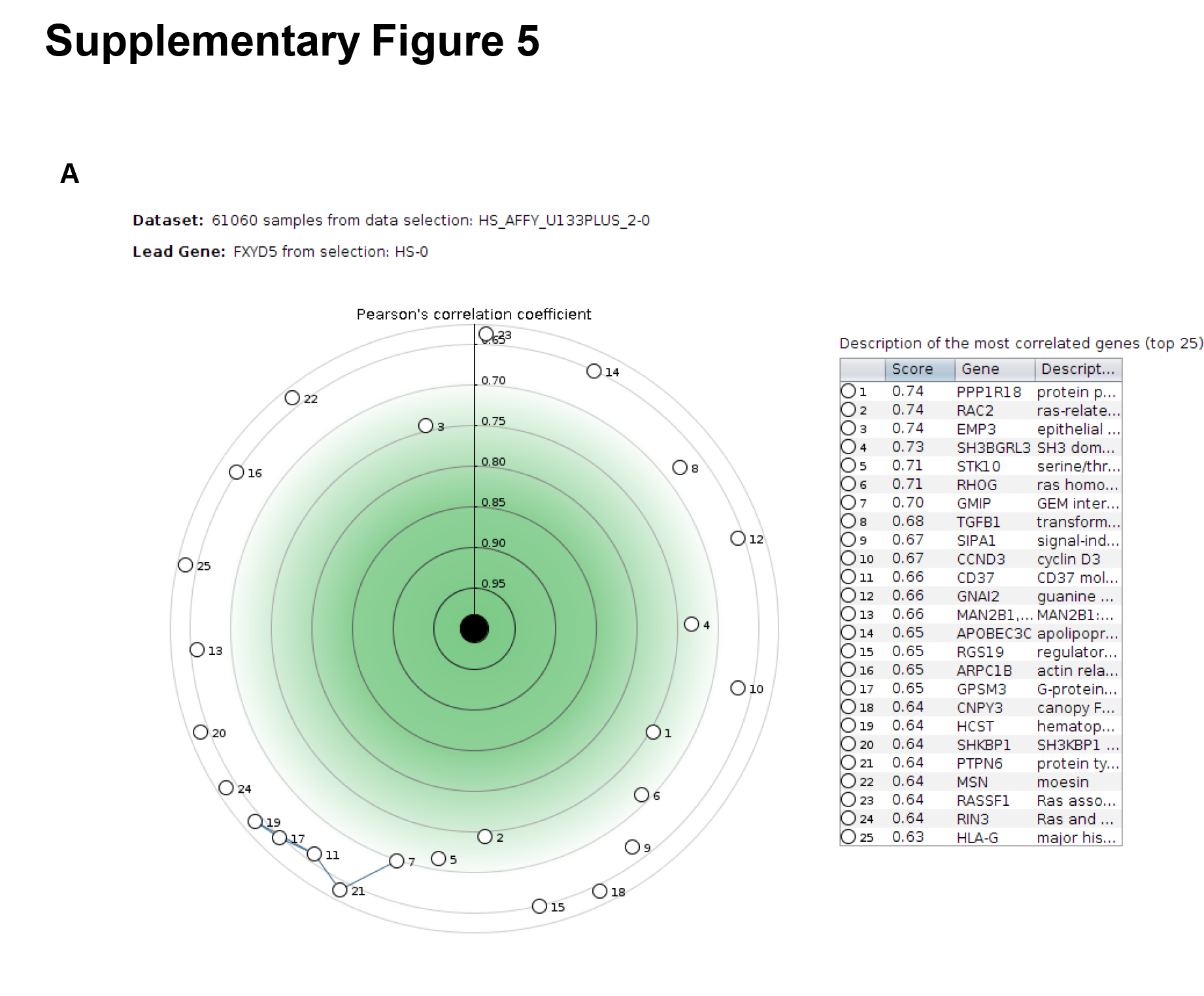

Supplement: Supplementary file 1 [file Data_Sheet_1.zip › SUPPL_FIGURE_5A_BESSO ET AL.tif]

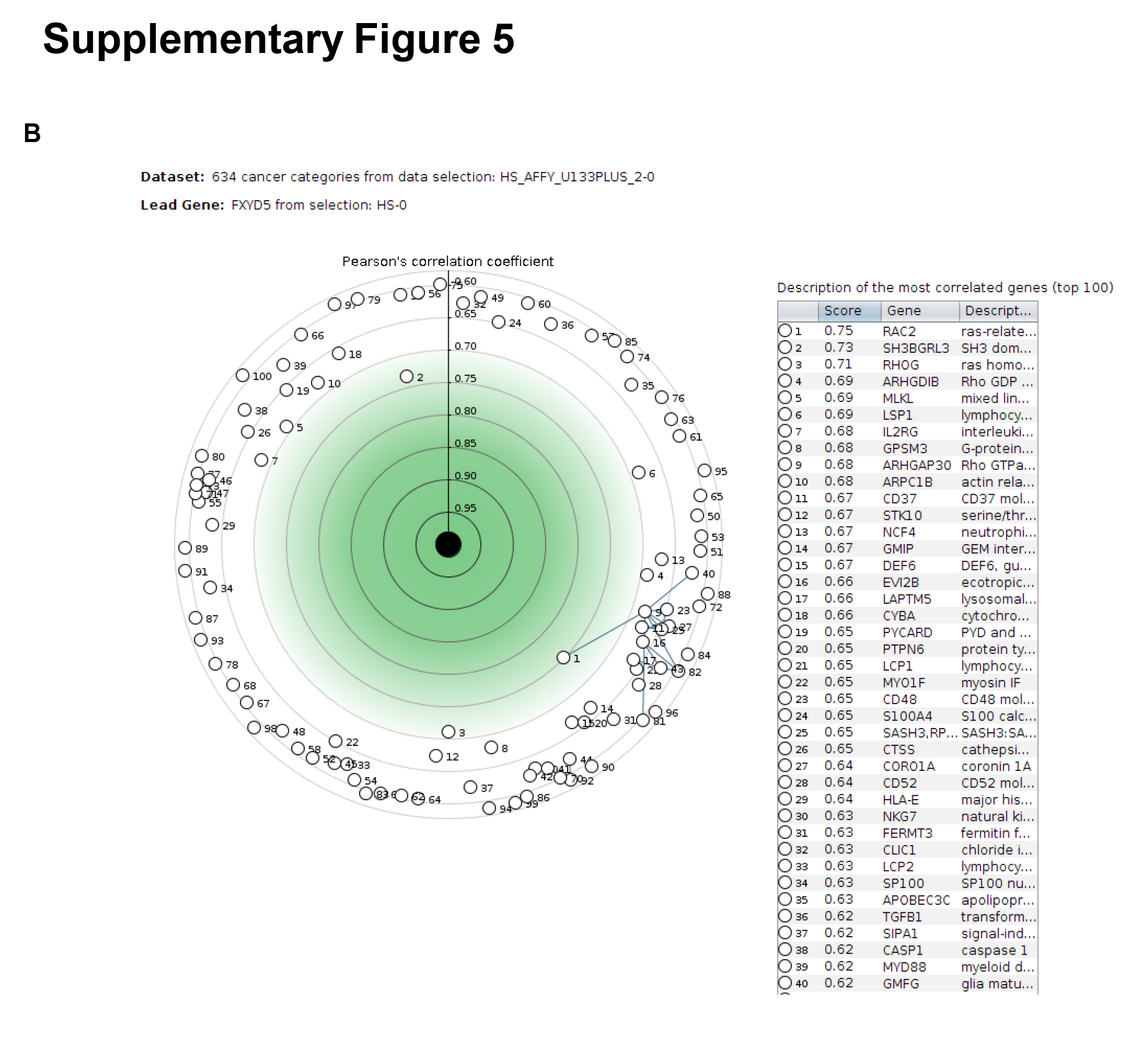

Supplement: Supplementary file 1 [file Data_Sheet_1.zip › SUPPL_FIGURE_5B_BESSO ET AL.tif]

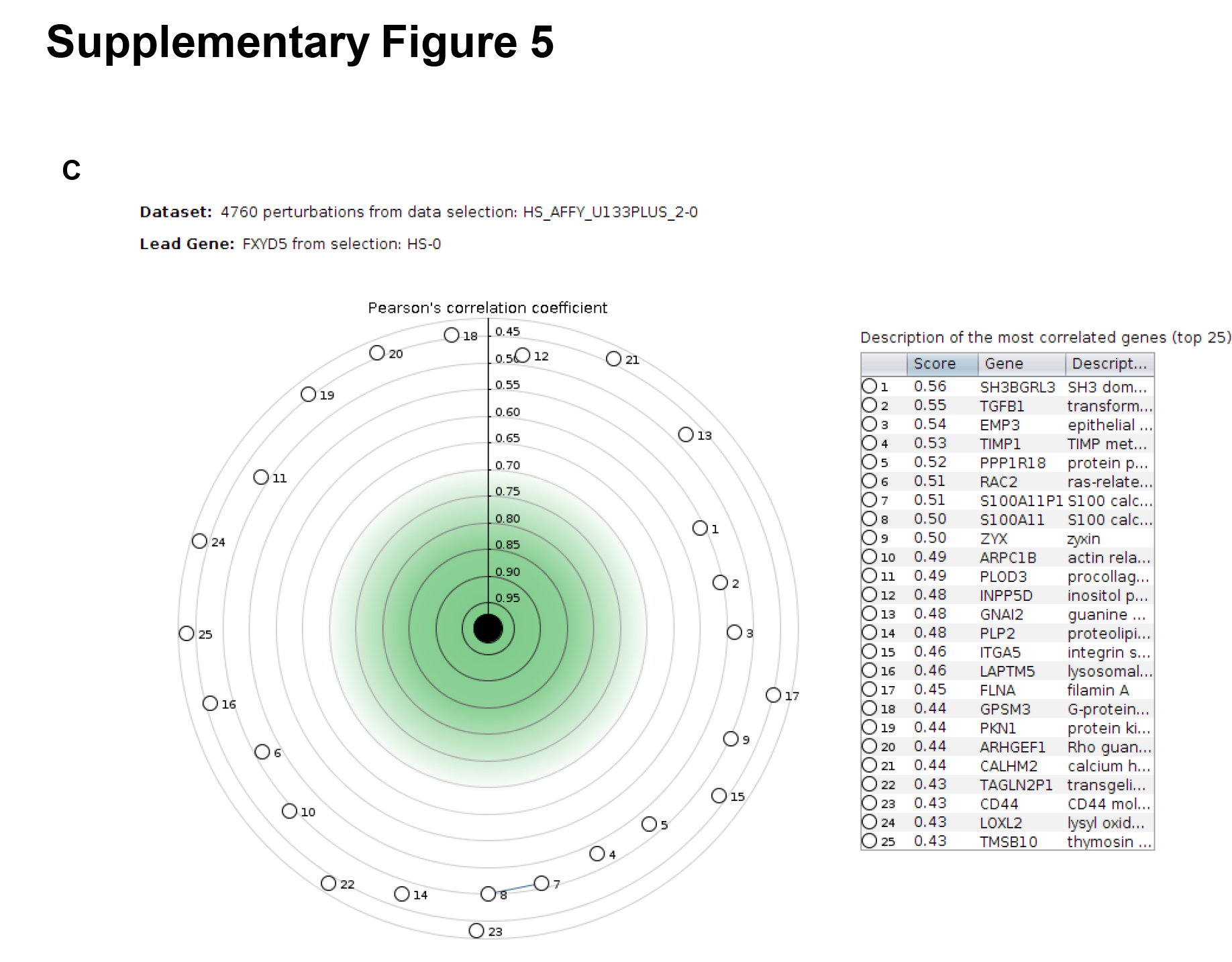

Supplement: Supplementary file 1 [file Data_Sheet_1.zip › SUPPL_FIGURE_5C_BESSO ET AL.tif]
