# Supplementary material for: FXYD5/Dysadherin, a Biomarker of Endometrial Cancer Myometrial Invasion and Aggressiveness: Its Relationship With TGF-β1 and NF-κB Pathways
Source: Front Oncol. 2019 Dec 6;9:1306. doi: 10.3389/fonc.2019.01306 (PMC6908519; doi:10.3389/fonc.2019.01306)
Supplement: Supplementary file 2 [file Data_Sheet_2.pdf]

Supplementary Figure 1. ROC curve analysis to evaluate diagnostic performance of FXYD5/Dys mRNA expression in UA. A. MI, B. Tumor grade, C. Risk of recurrence.

Supplementary Figure 2. A. RT-qPCR analysis of FXYD5/Dys expression in HGE cells transfected with 100 pmol FXYD5/Dys (HGE siRNA FXYD5/Dys) or scramble (HGE siRNA CTR) siRNA (***P<0.0001, Wilcoxon Signed Rank Test). B. Western immunoblotting of HGE siRNA FXYD5/Dys and HGE siRNA CTR and cell protein extracts using anti FXYD5/Dys monoclonal antibody (sc-166782; 2 µg/mL). C. Fluorescent immunocytochemistry of HGE siRNA FXYD5/Dys and HGE siRNA CTR cell monolayers using anti FXYD5/Dys polyclonal antibody (sc-98246; 2 µg/mL). Cell nuclei are visualized using HOESCHT 33342, bar: 20 µm.

Supplementary Figure 3. A. RT-qPCR analysis of FXYD5/Dys expression in Hec1a cells transiently transfected with pcDNA3 empty plasmid (Hec1a pcDNA3 cells) or pcDNA3-FXYD5/Dys plasmid (Hec1a pcDNA3-FXYD5/Dys cells) (***P<0.0001, Wilcoxon Signed Rank Test). B. Immunodetection of FXYD5/Dys by Western immunoblotting of Hec1a pcDNA3 and Hec1a pcDNA3-FXYD5/Dys cell protein extracts using anti FXYD5/Dys monoclonal antibody (sc-166782; 2 µg/mL)

Supplementary Figure 4. Correlation analysis of A. E-cadherin mRNA and protein levels (r= 0.3169, P<0.0001), B. E-cadherin mRNA and FXYD5/Dys mRNA (r=-0.3924, P<0.0001), C. E-cadherin protein and FXYD5/Dys mRNA (r=-0.1488, P=0.0030).

Supplementary Figure 5. Co-expression diagrams obtained using the Genevestigator bioinformatics tool, as a result of FXYD5/Dys gene expression profile analysis from datasets ‘SAMPLES’ (A.), ‘CANCER’ (B.) and ‘PERTURBATIONS’ (C.) using HS_AFFY_U133PLUS_2-0 platform (Affymetrix Human Genome U133 Plus 2.0 Array). TGF-β1 was ranked in positions 8, 36 and 2, respectively, in the previously mentioned datasets.
