# Supplementary material for: FXYD5/Dysadherin, a Biomarker of Endometrial Cancer Myometrial Invasion and Aggressiveness: Its Relationship With TGF-β1 and NF-κB Pathways
Source: Front Oncol. 2019 Dec 6;9:1306. doi: 10.3389/fonc.2019.01306 (PMC6908519; doi:10.3389/fonc.2019.01306)
Supplement: Supplementary file 3 [file Data_Sheet_3.pdf]

Supplementary Table 1. Clinicopathological parameters of EC tissue samples

Patient code	*Histology*	*FIGO stage*	*Grade*	*% MI*	*Risk group*
T1	Endometrioid	IA	1	<50%	Low
T2	Endometrioid	IA	1	<50%	Low
T3	Endometrioid	IA	1	<50%	Low
T4	Endometrioid	IA	1	<50%	Low
T5	Endometrioid	IA	1	<50%	Low
T6	Endometrioid	IA	2	<50%	Low
T7	Endometrioid	IA	2	<50%	Low
T8	Endometrioid	IA	2	<50%	Low
T9	Endometrioid	IA	2	<50%	Low
T10	Endometrioid	IA	2	<50%	Low
T11	Endometrioid	IA	2	<50%	Low
T12	Endometrioid	IA	2	<50%	Low
T13	Endometrioid	IA	2	<50%	Low
T14	Endometrioid	IA	2	<50%	Low
T15	Endometrioid	IA	2	<50%	Low
T16	Endometrioid	IA	2	<50%	Low
T17	Endometrioid	IA	2	<50%	Low
T18	Endometrioid	IA	2	<50%	Low
T19	Endometrioid	IA	2	<50%	Low
T20	Endometrioid	IA	2	<50%	Low
T21	Endometrioid	IA	2	<50%	Low
T22	Endometrioid	IA	2	<50%	Low
T23	Endometrioid	IA	2	<50%	Low
T24	Endometrioid	IA	2	<50%	Low
T25	Endometrioid	IA	2	<50%	Low
T26	Endometrioid	IA	3	<50%	Intermediate
T27	Endometrioid	IA	3	<50%	Intermediate
T28	Endometrioid	IA	3	<50%	Intermediate
T29	Endometrioid	IA	3	<50%	Intermediate
T30	Endometrioid	IA	3	<50%	Intermediate
T31	Endometrioid	IB	2	>50%	Intermediate
T32	Endometrioid	IB	2	>50%	Intermediate
T33	Endometrioid	IB	2	>50%	Intermediate
T34	Endometrioid	IB	2	>50%	Intermediate
T35	Endometrioid	IB	2	>50%	Intermediate

T36	Endometrioid	IB	2	>50%	Intermediate
T37	Endometrioid	IB	2	>50%	Intermediate
T38	Endometrioid	IB	2	>50%	Intermediate
T39	Endometrioid	IB	2	>50%	Intermediate
T40	Endometrioid	IB	2	>50%	Intermediate
T41	Endometrioid	IB	2	>50%	Intermediate
T42	Endometrioid	IB	2	>50%	Intermediate
T43	Endometrioid	IB	2	>50%	Intermediate
T44	Endometrioid	IB	2	>50%	Intermediate
T45	Endometrioid	IB	3	>50%	High
T46	Endometrioid	IB	3	>50%	High
T47	Endometrioid	IB	3	>50%	High
T48	Endometrioid	IB	3	>50%	High
T49	Endometrioid	IB	3	>50%	High
T50	Endometrioid	IB	3	>50%	High
T51	Endometrioid	IIA	1	>50%	High
T52	Endometrioid	IIA	2	>50%	High
T53	Endometrioid	IIA	2	>50%	High
T54	Endometrioid	IIB	1	>50%	High
T55	Endometrioid	IIB	2	>50%	High
T56	Endometrioid	IIB	2	>50%	High
T57	Endometrioid	IIB	2	>50%	High
T58	Endometrioid	IIB	2	>50%	High
T59	Endometrioid	IIB	2	>50%	High
T60	Endometrioid	IIB	3	>50%	High
T61	Endometrioid	IIB	3	>50%	High
T62	Endometrioid	IIB	3	>50%	High
T63	Endometrioid	IIIA	3	>50%	High
T64	Endometrioid	IIIC	3	>50%	High
T65	Endometrioid	IIIC	3	>50%	High
T66	Endometrioid	IIIC	3	>50%	High
T67	Clear cell	IB	3	>50%	High
T68	Clear cell	IV	3	>50%	High
T69	Endometrioid + Clear cell	IIIC	3	>50%	High
T70	Serous	IA	3	<50%	High
T71	Serous	IB	3	>50%	High
T72	Serous	IIIC	3	>50%	High
T73	Serous	IIIC	3	>50%	High
T74	Serous + Endometrioid	IB	3	>50%	High

Supplementary Table 2. Clinicopathological parameters of EC paired tissue samples from Superficial tumor and Invasive front

Patient code	***Histology***	***FIGO stage***	***Grade***	***% MI***	***Risk group***
T11 Superficial tumor	Endometrioid	IA	2	<50%	Low
T11 Invasive front	Endometrioid	IA	2	<50%	Low
T15 Superficial tumor	Endometrioid	IA	2	<50%	Low
T15 Invasive front	Endometrioid	IA	2	<50%	Low
T30 Superficial tumor	Endometrioid	IA	3	<50%	Intermediate
T30 Invasive front	Endometrioid	IA	3	<50%	Intermediate
T17 Superficial tumor	Endometrioid	IA	2	<50%	Low
T17 Invasive front	Endometrioid	IA	2	<50%	Low
T19 Superficial tumor	Endometrioid	IA	2	<50%	Low
T19 Invasive front	Endometrioid	IA	2	<50%	Low
T31 Superficial tumor	Endometrioid	IA	3	<50%	Intermediate
T31 Invasive front	Endometrioid	IA	3	<50%	Intermediate
T20 Superficial tumor	Endometrioid	IA	2	<50%	Low
T20 Invasive front	Endometrioid	IA	2	<50%	Low
T25 Superficial tumor	Endometrioid	IA	2	<50%	Low
T25 Invasive front	Endometrioid	IA	2	<50%	Low
T46 Superficial tumor	Endometrioid	IB	3	>50%	High
T46 Invasive front	Endometrioid	IB	3	>50%	High
T48 Superficial tumor	Endometrioid	IB	3	>50%	High
T48 Invasive front	Endometrioid	IB	3	>50%	High
T51 Superficial tumor	Endometrioid	IB	3	>50%	High
T51 Invasive front	Endometrioid	IB	3	>50%	High
T56 Superficial tumor	Endometrioid	IIB	2	>50%	High
T56 Invasive front	Endometrioid	IIB	2	>50%	High
T62 Superficial tumor	Endometrioid	IIB	3	>50%	High
T62 Invasive front	Endometrioid	IIB	3	>50%	High
T58 Superficial tumor	Endometrioid	IIB	2	>50%	High
T58 Invasive front	Endometrioid	IIB	2	>50%	High
T63 Superficial tumor	Endometrioid	IIB	3	>50%	High
T63 Invasive front	Endometrioid	IIB	3	>50%	High
T59 Superficial tumor	Endometrioid	IIB	2	>50%	High
T59 Invasive front	Endometrioid	IIB	2	>50%	High
T60 Superficial tumor	Endometrioid	IIB	2	>50%	High
T60 Invasive front	Endometrioid	IIB	2	>50%	High

T55 Superficial tumor	Endometrioid	IIB	1	>50%	High
T55 Invasive front	Endometrioid	IIB	1	>50%	High
T65 Superficial tumor	Endometrioid	IIIC	3	>50%	High
T65 Invasive front	Endometrioid	IIIC	3	>50%	High
T66 Superficial tumor	Endometrioid	IIIC	3	>50%	High
T66 Invasive front	Endometrioid	IIIC	3	>50%	High
T67 Superficial tumor	Endometrioid	IIIC	3	>50%	High
T67 Invasive front	Endometrioid	IIIC	3	>50%	High

Supplementary Table 3. Clinicopathological parameters of uterine aspirate EC biopsies

Patient code	*Histology*	*FIGO stage*	*Grade*	*% MI*	*Risk group*
AU1	Endometrioid	IA	1	<50%	Low
AU2	Endometrioid	IA	1	<50%	Low
AU3	Endometrioid	IA	1	<50%	Low
AU4	Endometrioid	IA	2	<50%	Low
AU5	Endometrioid	IA	2	<50%	Low
AU6	Endometrioid	IA	2	<50%	Low
AU7	Endometrioid	IA	2	<50%	Low
AU8	Endometrioid	IA	2	<50%	Low
AU9	Endometrioid	IA	2	<50%	Low
AU10	Endometrioid	IA	2	<50%	Low
AU11	Endometrioid	IA	2	<50%	Low
AU12	Endometrioid	IA	2	<50%	Low
AU13	Endometrioid	IA	2	<50%	Low
AU14	Endometrioid	IA	3	<50%	Intermediate
AU15	Endometrioid	IB	2	>50%	Intermediate
AU16	Endometrioid	IB	2	>50%	Intermediate
AU17	Endometrioid	IB	2	>50%	Intermediate
AU18	Endometrioid	IB	3	>50%	High
AU19	Endometrioid	IB	3	>50%	High
AU20	Endometrioid	IB	3	>50%	High
AU21	Endometrioid	IB	3	>50%	High
AU22	Endometrioid	IB	3	>50%	High
AU23	Endometrioid	IIB	1	>50%	High
AU24	Endometrioid	IIB	2	>50%	High
AU25	Endometrioid + Clear cell	IIIC	3	>50%	High
AU26	Serous	IIIC	3	>50%	High
AU27	Undifferentiated carcinoma	n/a	3	>50%	High
AU28	Heterologous Carcinosarcoma	IA	3	>50%	High

n/a: Not available

Supplementary Table 4. Primer list

Gene	*Forward 5´-3´*	*Reverse 5´-3´*	*bp*
**FXYD5/Dys**	CCAGCAACTGGAAGGAACGG	CGTCGTGGTGTCATCAGTGG	94
**FXYD5/Dys (cloning)**	AGTAAGCTTCTGATATGTCGCCCT CTGGTC	CGGATCCGAATTCTCACCTGCAACG ATTCC	542
**Snail**	CGTCTGCGGAACCTGCG	CGGTCAGCGAAGGCACG	120
**Slug**	TTCAACGCCTCCAAAAAGCC	GCGGTAGTCCACACAGTGAT	155
**ZEB1**	GAAAGTGATCCAGCCAAATGGAA	TTTGGGCGGTGTAGAATCAGAG	106
**CCL2**	TGCAATCAATGCCCCAGTCA	GTCTTGAAGATCACAGCTTCTTTG	126
**E-cadherin**	GACCAAGTGACCACCTTAGA	CTCCGAAGAAACAGCAAGAGC	172
**TNF-α**	GCCTGTACCCATGTTGTAGCAAA	TCTCTCAGCTCCACGCCATTG	101
**TNFR1**	ATTGGACTGGTCCCTCACCT	AGTAGGTTCCTTTGTGGCACTT	121
**TGF-β1**	AGTTGTGCGGCAGTGGTTGA	GCCGGTAGTGAACCCGTTGAT	121
**PAI-1**	CGGGGGTGGTGAACTCAGTGT	ACGCCTGGTGCTGGTGAATG	167
**GAPDH**	TGCACCACCAACTGCTTAGC	GGCATGGACTGTGGTCATGAG	88
**POLR2A**	TGGAGATCTTCACGGTGCTG	CAAGAGAGCCAAGTGTCGGT	116

Supplementary Table 5

Association between FXYD5/Dys, cytokines and chemokines mRNA expression levels in the TCGA UCEC dataset

Gene Expression		***FXYD5/Dys expression***		***P-value***
		Low	High	
CCL-2 expression	Low	87/137 (63.50%)	55/135 (40.74%)	<0.0001
	High	50/137 (36.50%)	80/135 (59.26%)	
TNF-α expression	Low	72/137 (52.55%)	58/135 (42.96%)	0.0048
	High	65/137 (47.45%)	77/135 (57.04%)	
IL-6 expression	Low	80/137 (58.39%)	68/135 (50.37%)	0.0053
	High	57/137 (41.61%)	67/135 (49.63%)	
IL-10 expression	Low	74/137 (54.01%)	65/135 (48.15%)	0.0013
	High	63/137 (45.99%)	70/135 (51.85%)	
CCL-17 expression	Low	88/137 (64.23%)	47/135 (34.81%)	0.0002
	High	49/137 (35.77%)	88/135 (65.19%)	
CCL-22 expression	Low	75/137 (54.74%)	36/135 (26.67%)	<0.0001
	High	62/137 (45.26%)	99/135 (73.33%)	
